# Similar somatotopy for active and passive digit representation in primary somatosensory cortex

**DOI:** 10.1002/hbm.26298

**Published:** 2023-05-05

**Authors:** Zeena‐Britt Sanders, Harriet Dempsey‐Jones, Daan B. Wesselink, Laura R. Edmondson, Alexander M. Puckett, Hannes P. Saal, Tamar R. Makin

**Affiliations:** ^1^ Wellcome Centre of Integrative Neuroimaging FMRIB, John Radcliffe Hospital Oxford UK; ^2^ Institute of Cognitive Neuroscience University College London London UK; ^3^ School of Psychology The University of Queensland Brisbane Australia; ^4^ Department of Psychology University of Sheffield Sheffield UK; ^5^ Queensland Brain Institute The University of Queensland Brisbane Australia; ^6^ MRC Cognition and Brain Sciences Unit University of Cambridge Cambridge UK

**Keywords:** finger representations, fMRI, hand map, motor, representational similarity analysis, somatosensation, topography

## Abstract

Scientists traditionally use passive stimulation to examine the organisation of primary somatosensory cortex (SI). However, given the close, bidirectional relationship between the somatosensory and motor systems, active paradigms involving free movement may uncover alternative SI representational motifs. Here, we used 7 Tesla functional magnetic resonance imaging to compare hallmark features of SI digit representation between active and passive tasks which were unmatched on task or stimulus properties. The spatial location of digit maps, somatotopic organisation, and inter‐digit representational structure were largely consistent between tasks, indicating representational consistency. We also observed some task differences. The active task produced higher univariate activity and multivariate representational information content (inter‐digit distances). The passive task showed a trend towards greater selectivity for digits versus their neighbours. Our findings highlight that, while the gross features of SI functional organisation are task invariant, it is important to also consider motor contributions to digit representation.

## Graphical abstract

Representation of the hand in primary somatosensory cortex was examined during active (movement) and passive (stationary stimulation) tasks. The representations these tasks produced were generally consistent in terms of location in the brain, somatotopic organisation, and inter‐digit representational structure. Some task differences were observed, including increased brain activity and increased multivariate representational content for the active task.

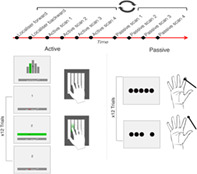

## 1 INTRODUCTION

Traditionally, the organisation of the somatosensory system has been studied using passive tactile stimulation protocols, that is, contacting stationary digit(s) with tactile stimuli (examples in humans: (Besle et al., 2014; Sanchez‐Panchuelo et al., 2010) and in primates: (Michael M. Merzenich et al., 1978; Sur et al., 1980)). While providing a highly controlled means to study the primary somatosensory cortex (SI), active touch tasks (Kikkert et al., 2016; Schellekens et al., 2018), involving movement of the digits, may provide somatosensory inputs more like those that occur habitually. In daily life, the majority of hand inputs to SI occur from deliberate action (i.e., driven by motor control), or are directly relevant for supporting motor control (Scott, 2004). Accordingly, somatosensory hand representation reflects habitual patterns of coordinated action between digits (Ejaz et al., 2015; Ingram et al., 2008). These usage effects manifest in greater overlap between digit representations (e.g., multi‐digit receptive fields) for digits used more frequently together in daily life (Ejaz et al., 2015; M. M. Merzenich et al., 1984). Indeed, there is growing recognition that somatosensory and motor processing are intimately and bi‐directionally linked (Aronoff et al., 2010; Gomez et al., 2021; Mao et al., 2011; Rathelot et al., 2017; Wasaka et al., 2021). For example, accumulating evidence suggests an important role for SI in motor processing (Brecht, 2017), correction of ongoing movements (Pruszynski et al., 2016), and motor learning (Darainy et al., 2013; Kumar et al., 2019; Mathis et al., 2017). Preparatory activity has been shown in SI during motor planning, that is, before afferent information is available (Ariani et al., 2022; Gale et al., 2021), and SI stimulation can modulate motor behaviour via direct spinal projections (Karadimas et al., 2020; Matyas et al., 2010).

Crucially, there is a strong prediction that the fine‐grained features of digit representation identified under active conditions would vary from the traditional features established using passive paradigms. First, muscle proprioceptors and skin stretch receptors will typically be more activated in active than passive tasks (Bensmaia & Tillery, 2014; Chouvardas et al., 2008; Saal et al., 2017). Biomechanical enslaving of the digits (Lang & Schieber, 2004; Reilly & Schieber, 2003) could interfere with selective movement of single digits in active touch, reducing individuated representation in the brain. Sensory prediction (efference copy) from the motor system (Wolpert et al., 1995; Wolpert & Flanagan, 2001) could cause additional SI excitation (or inhibition through sensory gating: London & Miller, 2013) during active tasks. Moreover, several other high‐order cognitive processes, which may play a role during active exploration, have been shown to influence SI representation, including attention (Eimer et al., 2001; Puckett et al., 2017), reward (Pleger et al., 2008), and visual input (Kuehn et al., 2014). Alternatively, since these divergent inputs will ultimately converge into the same SI neural network and feed into somatotopically restricted locations (see the ‘*Mechanisms underpinning representational consistency between tasks*’ section in the *Discussion*), it could be argued that the resulting canonical representational features should be essentially preserved, regardless of whether SI is being activated by movement or passive touch.

Few studies, however, have directly compared whether key features of somatotopy remain consistent between active and passive tasks. Recent representational similarity analysis (RSA) by Berlot et al. (2019) showed that when task and stimulus properties are tightly matched, the representational structure of the hand representation does not vary across tasks. They did note some task differences, however, with greater overall activity in the active task and a trend towards increased information content under passive conditions (characterised as increased inter‐digit multivariate pattern dissimilarity). Nevertheless, since the multivariate measures used by Berlot are naïve to spatial relationships, these findings do not directly inform on several hallmark organisation properties of SI which have been the focus of most previous studies (e.g., Besle et al., 2014; Kolasinski et al., 2016).

Here, we compared spatial and multivariate SI features under active and passive digit tasks that are prominently featured in functional magnetic resonance imaging (fMRI) studies, using available data from a previous 7 Tesla fMRI study (D. B. Wesselink et al., 2022). First, we explored spatial correspondence in the location of SI digit maps between the active and passive task (Section 3.1). Second, we compared somatotopic organisation between tasks (Section 3.2). Third, we looked at the multivariate representational structure of the hand between tasks (Section 3.3). Because the parameters of the tasks we examine here were not matched (as in Berlot et al., 2019), this allowed some insight into whether SI representation may diverge under distinct stimulation and task demands, as is typical across different experiments. We predicted major topographic features of digit somatotopy would be consistent between tasks because, despite differing types or amount of information, all inputs feed into somatotopically restricted regions (Kuehn et al., 2017; Qi & Kaas, 2004). To explore whether peripheral differences across tasks are sufficient to explain the observed differences between tasks, we employed a computational model (Touchsim: Saal et al., 2017) that simulates SI hand representation. Using this model, we specifically tested whether differences between tasks could be explained by either increased overall activity (gain modulation) in the active task, or by digit enslavement during the active task which could increase the co‐activation of digits.

## 2 MATERIALS AND METHODS

### 2.1 Participants

Fifteen healthy volunteers (six females, age ± SEM = 26.44 ± 1.04) were recruited for this study. All participants except one were right‐handed and all experimental tasks were performed using the right hand. The fMRI session was part of a larger study, full information and study protocol can be found at https://osf.io/nh4yp/ and in Wesselink et al. (2022). All participants gave written informed consent and ethical approval for the study was obtained from the Health Research Authority UK (13/SC/0502). One (right‐handed) participant was excluded as an outlier from the passive task multivariate analysis (RSA; Section 3.3). This was done as the correlation between this individual's hand representational dissimilarity matrix (RDM; an RSA output measure) and the group average RDM for the passive task was >3σ below the group mean; that is, that individual's multivariate data showed very low representational ‘typicality’ and was, thus, considered an outlier.

### 2.2 MRI tasks

#### 2.2.1 General procedure

**FIGURE 1:**
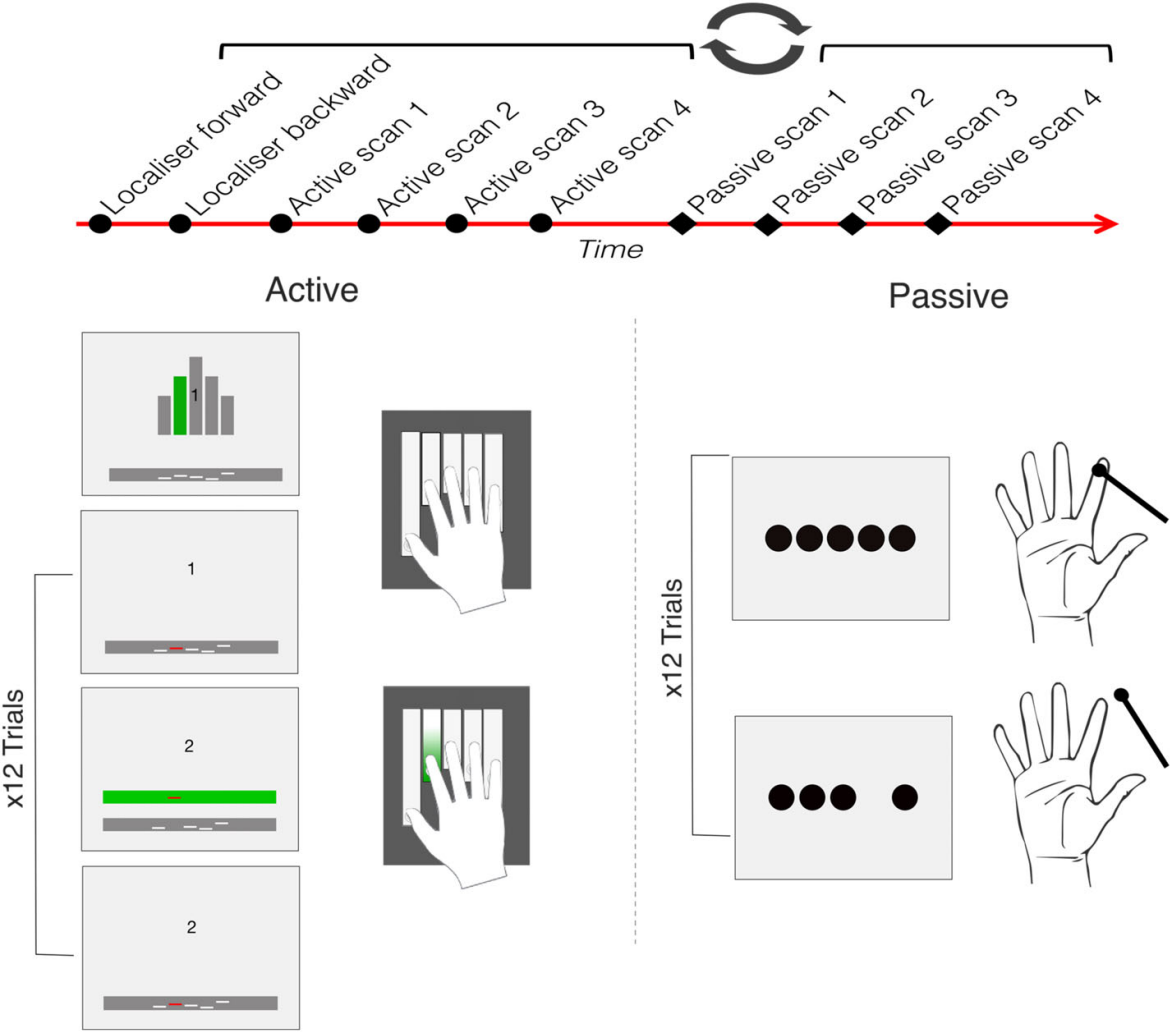
Overview of the two tasks used in this study. *Top*: Participants completed an active and a passive task inside the scanner (order of tasks was counterbalanced across participants). Each task consisted of four consecutive scans (two active, two passive). The active task was always preceded by two functional localiser scans. *Left*: Example of one block of the active task (trials repeated 12 times). During the active task, participants were required to press keys on an MRI‐safe keyboard with their right hand. Five white lines were presented within a grey box near the bottom of the screen, representing the five digits at rest. An instruction display indicated the digit to be used in the upcoming block (the ‘target’ digit) was highlighted in green on a hand schematic (top), then the white line representing the target digit turned red. For each trial, participants were informed that whenever the green box appeared above the grey box, they were to press the key and move the red line into the green box and hold it there for 400 ms before releasing. Twelve such trials were completed per digit at a rate of ~1 Hz in a block before the instruction display for the next block was shown. Participants were awarded points for successfully completing each trial. *Right*: Example of one block of the passive task (trials repeated 12 times). In the passive task, participants rested their right hand in a supine position on a foam support. Participants had their individual digits tapped by the experimenter at a rate of 1 Hz using a plastic probe. At the same time, they saw five dots displayed on the screen corresponding to their five digits and the dot corresponding to the stimulated digit would flash on and off at the same time as the stimulation was occurring. Each digit was tapped 12 times in a block.

Participants completed an active and a passive task during a single session of ultra‐high field (7 Tesla, ‘7 T’) fMRI. There were four consecutive scans per task (Figure 1 top; order of active vs. passive scans counterbalanced between participants), with each scan lasting ~4.5 min. The scan structure was identical for the active and the passive task. Each scan consisted of a pseudo‐randomised block design with 15 experimental blocks in total (3 blocks per digit) interspersed with three 12–24 s rest blocks. Each experimental block contained 12 single‐digit trials. For the passive task, these trials consisted of 12 taps at ~1 Hz (see *Passive task* section), and for the active task these trials consisted of 12 movements at a rate of ~1 Hz (see *Active task* section).

In addition to the two main experimental tasks, a functional localiser was carried out before the active task to identify digit‐specific clusters (or regions of interest). This allowed us to create clusters independently from our main tasks, which were used (as masks) to probe univariate somatotopic neighbourhood relationships (Section 3.2.2). This localiser was comprised of two scans lasting ~4 min each, with a set inter‐digit sequence block design and no rest blocks (see *Digit‐specific clusters localiser* section (also see Kikkert et al., 2016; Kolasinski et al., 2016; Mancini et al., 2012; Sanchez‐Panchuelo et al., 2010; Wandell et al., 2007; Zeharia et al., 2015)).

#### 2.2.2 Active task

During the active task, participants were required to move individual digits by making key presses on an MRI‐safe custom‐made keyboard (Berlot et al., 2019; Ejaz et al., 2015; D. B. Wesselink et al., 2019; Xu et al., 2017). Under each key, there was a force transducer which measured the force applied by each digit. The digit to be moved in the upcoming block (the ‘target’ digit) was indicated by an instruction display, where one of five grey bars was in highlighted green (see Figure 1, left). In each trial participants were required to move a cursor from the starting position (inside a grey box at the lower part of the screen) into a green box that appeared above the grey box. This was achieved by pressing the keyboard key under the target digit. To promote task engagement and encourage participants to perform the task well, participants received a point for successful completion after hovering the cursor inside the box for 400 ms. They were then instructed to release the force on the keyboard key—allowing the cursor to fall back into the starting position grey box, ready for the next trial. Each trial lasted ~1 s and there were 12 trials (i.e., 12× digit movements) per block before the instructions for the next digit block were shown. There were 15 blocks in total (3 blocks per digit) in each scan. The keys required force application to activate but the keys were not movable; therefore, the actual movement of the digit was low (~1 mm).

#### 2.2.3 Passive task

In the passive stimulation task, participants were asked to rest their right hand in a comfortable, supine position on a foam support. A trained experimenter used a plastic probe to tap the distal pad of a single digit at a 1 Hz rate, before moving on to the next digit. There were 12 trials (taps) per digit in each digit block before the experimenter moved on to the next digit, and 15 blocks per scan (three per digit). Timing was controlled by audio cues presented to the experimenter over headphones. During the task, participants were shown five white dots on the screen, corresponding to each of the five digits (Figure 1, right). One dot, indicating the stimulated digit, would flash at a rate of 1/s whilst the digit was being stimulated to help participants attend to that digit (note that attention is known to modulate digit representations in SI; Puckett et al., 2017). To promote engagement during the task, double taps were administered randomly (one double‐tap per digit condition in each scan). Participants were asked to indicate when they felt the double taps by pressing a button on a button‐box placed in their left hand.

#### 2.2.4 Digit‐specific clusters localiser

An independent functional localiser was carried out using a traveling wave design task in order to identify digit‐specific clusters used for later analysis. Participants used the same keyboard and visual display as in the active task (see Section 2.2.2). Two separate scans with reverse orders, forward (digit 1‐2‐3‐4‐5) and backward (digit 5‐4‐3‐2‐1) cycles, were used to overcome potential order‐related biases due to the sluggish hemodynamic response (Besle et al., 2013).

In each scan, the cycle was repeated five times continuously with no rest periods in between. Each cycle consisted of five blocks (one per digit) which progressed in either the forward or backwards order of digits. At the start of each block, participants were visually instructed which of their digits to use in the upcoming block (see Figure 1). Eight trials were then completed in which the participant had to move one of their digits eight times at 1 Hz, before the instructions for the next digit was shown. Each scan lasted ~4 min.

### 2.3 MRI acquisition

All MRI measurements were acquired using a Siemens 7 T Magnetom scanner with a 32‐channel head coil. Task fMRI data were acquired using a multiband GE echo planar imaging (EPI) sequence with an acceleration factor of 2 (Moeller et al., 2010; Ugurbil et al., 2013). A limited field‐of‐view (FOV) was used consisting of 56 slices each 1 mm thick over the primary somatosensory cortex with a 192 × 192 mm in‐plane FOV (TR: 2000 ms, TE: 25 ms, FA: 85°, GRAPPA factor: 3). This resulted in spatial resolution of 1 mm isotropic. A whole brain anatomical T1‐weighted (MPRAGE) image was also collected with a 1 mm isotropic spatial resolution (FOV: 192 × 192 × 176, TR: 2200 ms, TE: 2.82 ms, FA: 7°, TI: 1050 ms, GRAPPA factor: 4).

### 2.4 MRI pre‐processing

All MRI data pre‐processing and analysis was carried out using FMRIB Software Library (Jenkinson et al., 2012; FSL, version 6.0) as well as MATLAB scripts (version R2014b) which were developed in‐house. Surface reconstruction was carried out using FreeSurfer (Dale et al., 1999; www.freesurfer.net, version 6) and results from the task and travelling wave analysis were projected onto the cortical surface for visualization purposes using Connectome Workbench software (Marcus et al., 2011; www.humanconnectome.org, version 1.2.3).

#### 2.4.1 Pre‐processing

Standard pre‐processing steps were carried out using FSL. FSL's Expert Analysis Tool (FEAT) was used to carry out motion correction (using MCFLIRT; Jenkinson et al., 2002), brain extraction (BET; Smith, 2002), spatial smoothing using a 1 mm full width at half maximum Gaussian kernel (as in Wesselink et al., 2022) and high‐pass filtering using a cut‐off of 100 s. The output from the MCFLIRT analysis were visually inspected for excessive motion (defined as >1 mm absolute mean displacement). No participants had an absolute mean displacement greater than 1 mm.

#### 2.4.2 Image registration

All analyses were carried out in the participants' native space. For each participant, a mid‐space was calculated between the four active and four passive scans, that is, the average space in which the images are minimally reoriented. Each scan was then aligned to this session mid‐space using FMRIB's Linear Image Registration Tool linear registration (FLIRT; Jenkinson et al., 2002; Jenkinson & Smith, 2001). This registration was also run separately for the functional localiser (travelling wave) scans, where the forward and the backward scans were realigned to their mid‐space. The localiser mid‐space was then registered to the mid‐space of the active and passive tasks using FLIRT. Finally, as these scans were collected as part of a larger project containing two scanning sessions (https://osf.io/nh4yp/), the mid‐spaces of the two scanning sessions were aligned together into a study mid‐space (please note: only day 1, the baseline session, of this larger dataset was analysed for the purposes of this article).

To ensure an accurate co‐registration of the hand‐knob (Yousry et al., 1997), manual adjustments to the translations and rotations were carried out in SPM (https://www.fil.ion.ucl.ac.uk/spm/; version 12) This was done by overlaying the EPI on the T1‐weighted image and visually optimising the match of the boundaries and contours of the hand‐knob. Once an accurate registration was achieved, a final mid‐space was calculated to which all scans were re‐aligned.

### 2.5 Initial analysis

#### 2.5.1 Active and passive tasks

To identify activity patterns for each digit condition, a voxel‐based general linear model (GLM) analysis was carried out on each active/passive scan using FEAT. For the active task, the average force output from the keyboard per TR was modelled, whereas for the passive task a 12 s stimulation block was modelled. The design was convolved with the double‐gamma haemodynamic response function, as well as its temporal derivative. The force output could not be read for one run of participant 7. The GLM for this participant/run was instead modelled using the (convolved) instructed trial sequence. Eleven contrasts were set up: each digit versus rest, each digit versus all other digits and all digits versus rest. The estimates from the four active/passive scans were then averaged voxel‐wise using a fixed effects model with a cluster forming z‐threshold of 3.1 (Eklund et al., 2016) and family‐wise error corrected cluster significance threshold of *p* < .05.

#### 2.5.2 Creation of digit‐specific clusters (regions‐of‐interest)

The identification of digit‐specific voxel clusters from the independent localiser (used for the analysis of somatotopic neighbourhood relationships, see Section 3.2.2) was carried out as in Kikkert et al. (2016). In short, a reference model was first created using a convolved hemodynamic response function to account for the hemodynamic delay. This model consisted of an 8 s ‘on’ period followed by 32 s ‘off’ period to model the movement block of one digit for one cycle. The model was shifted 20 times by one lag of 2 s (runs were acquired with a TR of 2 s) to model one entire movement cycle (which lasted 40 s). This resulted in 20 reference models, and was repeated five times to model the five cycles in each scan. Following this, the pre‐processed BOLD signal time course for each voxel was correlated with each of the reference models. This resulted in cross correlation *r*‐values, which were standardized using the Fisher's r‐to‐z transformation. Lags were assigned to each digit (four lags per digit) to average the *r*‐values across scans for each voxel. This resulted in an *r*‐value for each digit, which was further averaged across the forward and backward runs. Each voxel was assigned to one digit using a ‘winner‐takes‐all’ approach. This was done by finding the maximum correlation for each voxel across the five averaged values and assigning the voxel to the digit with max correlation.

**FIGURE 2:**
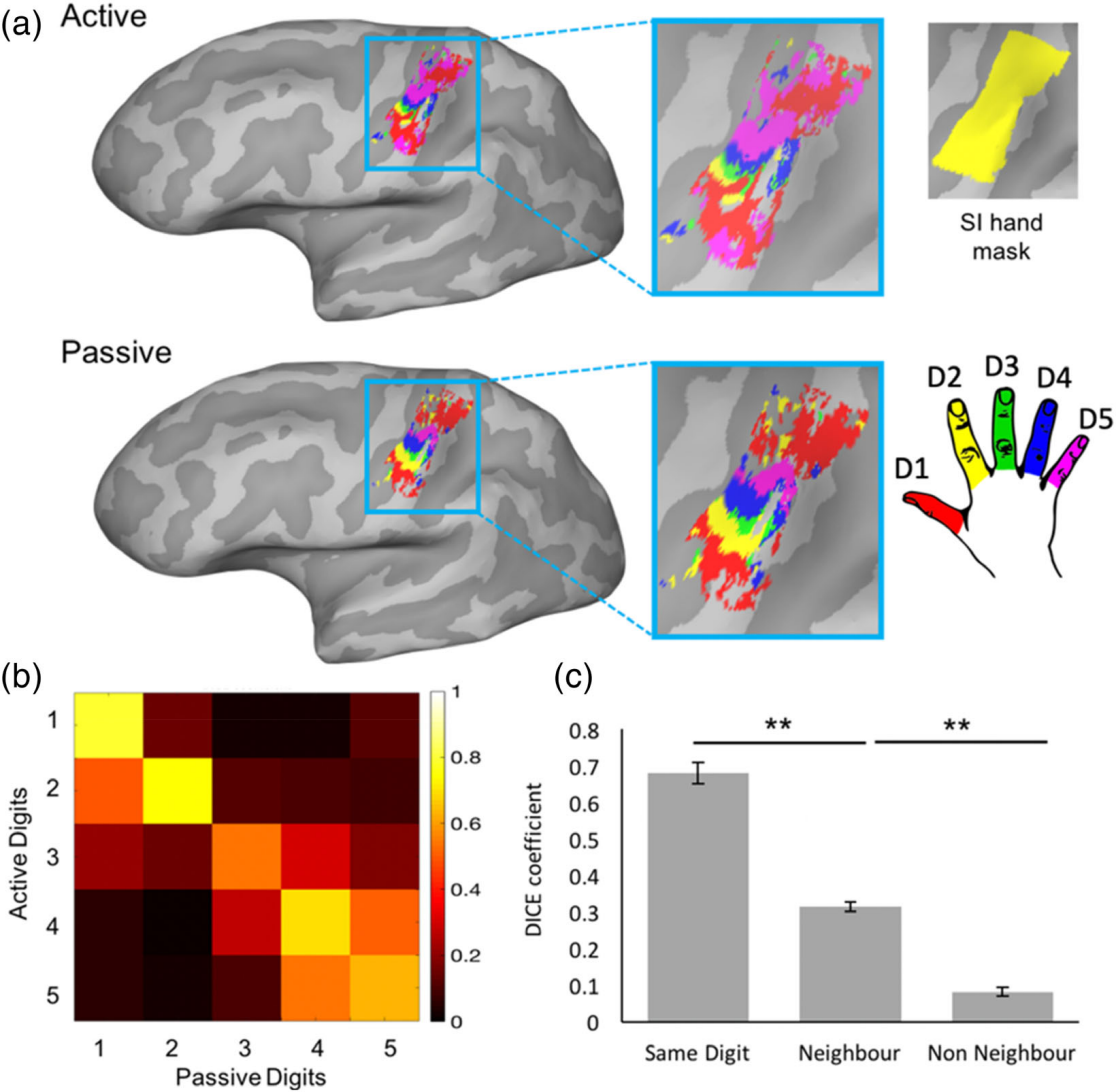
Spatial correspondence between active and passive tasks. (a) For an example participant, the minimally thresholded activity for both tasks is shown projected onto the cortical surface (red = D1, yellow = D2, green = D3, blue = D4, pink = D5). Activity was masked using an anatomically defined tight SI hand mask including Brodmann areas 3a, 3b, and 1, based on FreeSurfer segmentation, shown in inset. (b) 5 × 5 matrix showing spatial overlap in the SI hand mask between digits/tasks as measured by the Dice coefficient, averaged across all participants. The diagonal represents the average overlap of the same digits, whereas the off‐diagonal elements represent average overlap between neighbouring and non‐neighbouring digits. Hotter colours indicate greater overlap (see legend). (c) Bar chart showing average overlap for the same digits (averaged values across the diagonal line in (b)), neighbouring digits (averaged values of the off‐diagonal in (b)), and non‐neighbouring digits across the tasks (all other cells in (b)). There was greater spatial overlap for the same digit across tasks than for neighbouring digits across tasks. Similarly, spatial overlap was greater for neighbouring digits across tasks than non‐neighbouring digits (measured with *t* tests). Error bars show standard error of the mean. Significance is indicated by: ** = *p* < .001, * = *p* < .05.

**FIGURE 3:**
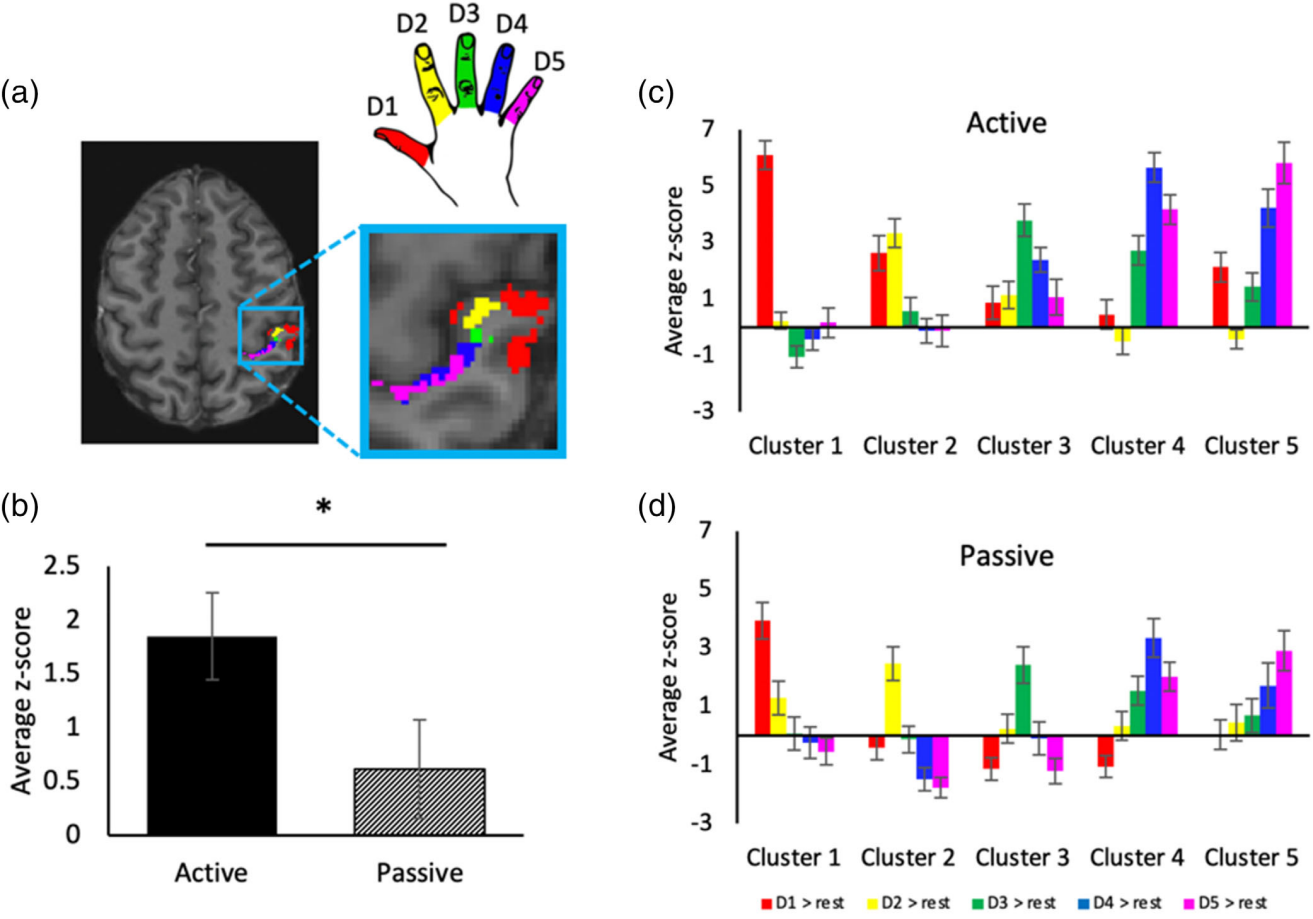
Neighbourhood relationships between activity patterns within digit‐specific clusters for the active and passive tasks. (a) Example of digit‐specific clusters created using the independent localiser for one participant, approximately located in BA3b. Activity for each digit (vs. rest) was extracted within each digit‐specific cluster, done separately for the active and passive tasks. (b) Overall average activity levels were significantly higher in the active task than in the passive task. (c, d) For both tasks, a somatotopic activity gradient can be seen within each digit‐specific cluster: with the target digit producing on average the greatest activity within its own digit‐specific cluster, followed by its neighbouring digits, and lowest activity seen in non‐neighbouring digits. * indicates significant differences at *p* < .05.

To correct for multiple comparisons, a false discovery rate (FDR; Benjamini & Hochberg, 1995) threshold (*q* < 0.01) was applied to each digit individually (Kikkert et al., 2016). The resulting FDR corrected digit‐specific voxels were then used to create digit‐specific clusters. This was done by using an anatomically defined mask of the hand region (SI hand mask, Figure 2a inset) which was defined for each participant based on a FreeSurfer probabilistic structural segmentation of SI subdivisions (thresholded at 95%). Brodmann Areas 3a, 3b, and 1, spanning a 2 cm strip medial/lateral to the anatomical hand knob were included in the mask (D. B. Wesselink et al., 2019). Area 2 was not explicitly included as a probabilistic marker of SI due to its overlap with posterior parietal area 5 in the FreeSurfer segmentation. Due to the probabilistic nature of the segmentation, however, the resulting mask also incorporated considerable parts of area 2 due to the overlap of areas 1 and 2 in the area 1 segmentation (see figure S4 in Wesselink et al., 2022). The digit specific activity within this mask was used to create the digit‐specific clusters (see, e.g., Figure 3a).

### 2.6 Key analyses

Data from the active and the passive task were analysed in three different ways to probe different hallmark aspects of digit representation. First, we investigated correspondence in spatial location between digit maps obtained in the active and passive tasks (Section 3.1). Second, we examined somatotopic organisation with the somatotopic index analysis (Section 3.2.1). Within each digit specific cluster (as identified by the functional localiser) we also examined the somatotopic neighbourhood activity for each digit using traditional univariate analysis (Section 3.2.2). Finally, we examined the multivariate representational structure of the hand using (unthresholded) activity patterns across the SI hand area using RSA (Section 3.3). All data used for final analysis will be made available online at the Open Science Framework upon publication of this manuscript (www.osf.io/nh4yp).

#### 2.6.1 Spatial correspondence

To assess the spatial correspondence between digit representations in the active and passive tasks (Section 3.1), a Dice coefficient (Dice, 1945) was calculated on activity maps projected onto the cortical surface (as in Kikkert et al., 2016) for each individual participant. Traditionally, the amount of spatial overlap between, in this case, two digit representations, is calculated in reference to the total spatial area of these two digits. However, cases may occur when one digit's representation is completely within another's (i.e., completely overlapping), but the area of the second digit may be much larger than the first, leading to a small Dice coefficient. To account for these differences, the Dice coefficient was normalised to the smallest area of the pair of digits (see Formula 1). This constrains the maximum overlap to be equal to the smallest digit representation area. Note: the same analysis was run with the traditional Dice coefficient with comparable results. This analysis can be found in the Supplementary Materials, Section 1, and Figure S2.

$$\frac{\left|A\cap B\right|}{\min\left(\left|A\right|,\left|B\right|\right)}$$

*Formula 1*. ‘*A*’ represents the spatial area of one digit representation and ‘*B*’ the spatial area of another. This produces values of spatial overlap ranging from 0 to 1, with 0 indicating no spatial overlap and 1 representing perfect overlap.

For each digit, the activity maps from the active and passive task (contrasting each digit vs. all the other digits; see Section 3.5.1) were projected onto the cortical surface, were minimally thresholded (*Z* > 2; Kikkert et al., 2016) on the cortical surface and masked using the BA1 and 3 SI hand mask described above (see Figure 2a and Figure S1 for all participants). Dice values were computed across tasks and digits resulting in a 5 × 5 matrix (see Figure 2b), with the diagonal representing the Dice coefficient of the same digits across active and passive tasks. The unit of spatial area in this analysis is the number of nodes (of the 2D parcellation of the cortical surface). Given the analysis is performed on the surface, these nodes follow the individual anatomical features of our participants. This, therefore, means we are comparing spatial relationships while taking curvature and anatomy into account.

Given the topographic features of the digit map, we should see more overlap when comparing the same digits across tasks, than when comparing neighbouring digits across tasks. Additionally, more overlap should be observed when comparing neighbouring digits across tasks than when comparing non‐neighbouring digits. Paired‐samples *t* tests were carried out to determine whether this was the case across participants. Alpha levels were Bonferroni‐adjusted to account for these two comparisons (*p* < .025).

#### 2.6.2 Somatotopic organisation

We next looked at single‐digit activity profiles to investigate whether the digit representations followed a comparable somatotopic organisation between tasks (Section 3.2). To explore this, we first performed an analysis of whether the digit specific clusters sat in a somatotopic line along the dorsomedial‐ventrolateral axis, that is, to what extent the expression of digit preference followed the expected somatotopic order (see the ‘*somatotopic index*’ analysis, Section 3.2.1). We then looked within these digit specific clusters at the activity when stimulating each digit, examining whether a topographic pattern could be observed, that is, whether activity decreased for stimulation of digits further from the ‘target’ digit (the digit of that cluster). This analysis was referred to as the ‘*neighbourhood relationships*’ analysis (Section 3.2.2).

In more detail, for the somatotopic index analysis (Section 3.2.1), we assigned each node (i.e., voxel on the surface of the brain) a value indicating its digit preference. This was calculated as the average of digit identity (1–5), weighted by their respective z‐transformed activity against rest. For example, if the z‐statistic is 3 for digit 2 (D2) and D3, but 0 for the other digits, the resulting index is 2.5. Next, these indices were correlated with the nodes' dorsomedial‐ventrolateral positions along SI. We used circular correlation as described by Jammalamadaka and Sengupta (2001):

$$r_{\text{circ}}=\sum_{k=1}^{n}\left(\sin\left(a_{1k}-\mu_{1}\right)∙\sin\left(a_{2k}-\mu_{2}\right)\right)/\sqrt{\sum_{k=1}^{n}\sin^{2}\left(a_{1k}-\mu_{1}\right)∙\sum_{k=1}^{n}\sin^{2}\left(a_{2k}-\mu_{2}\right)}$$

where *ɑ*_*pk*_ are the values from group *p* transformed to radians and *μ*_*p*_ is the group mean. This metric essentially tests whether moving ventrolaterally along SI coincides with an ulnar (D1–D5) rotation along the hand, that is, somatotopic organisation. A more somatotopic organisation is indicated by higher correlation values. The use of circular correlation was deemed to be preferable to regular correlation methods in this instance due to the frequent presence of ‘double‐thumb’ representations, that is, the presence of two maps of the thumb in the SI hand map, typically with a D1 cluster ventrally to the D5 cluster (Kikkert et al., 2016). The correlational coefficients for the active and passive task were compared using a paired *t* test. This allowed us to determine whether somatotopic organisation was more pronounced in one condition compared to the other. That is, whether the correlation was significantly higher in one condition.

For the neighbourhood relationships analysis (Section 3.2.2), we looked at activity patterns for each digit within each digit specific cluster (identified with the independent localiser, see Figure 3a for an example) versus rest. For each digit, the activity versus rest was extracted and then averaged for each digit specific cluster (Figure 3c,d). Data were shifted above zero to remove any negative values by subtracting the lowest value for each participant.

Within each digit‐specific cluster, we then calculated the difference in activity between the target digit and its neighbours by subtracting the neighbour digit activity value from the target digit. This difference was then divided by the target digit activity level, for example, in cluster 3: ([D3‐D2]/D3). This was repeated for the target digit and non‐neighbours, for example, in cluster 3: ([D3‐D5]/D3). Therefore, larger values mean there is a greater difference in activity between the target digit and the neighbouring or non‐neighbouring digits. The analysis was performed separately for each task. If activity follows the expected somatotopic neighbourhood relationships, activity differences should be greater when comparing the target digit and its non‐neighbours, than comparing the target digit and its immediate neighbours. Note: for this analysis, D5 was considered as a non‐neighbour to D1. Due to the prominence of double‐thumb representations (see above), this analysis was repeated with D5 as a neighbour to D1—see Supplementary Materials, Section 3.

Differences in these neighbourhood relationships (i.e., activity differences between neighbouring or non‐neighbouring digits) was compared across the two tasks using a repeated measures ANOVA. Significant interactions were followed up using post hoc tests. ANOVA has previously been found to be relatively robust to violations of normality (Glass, 1972; Harwell, 1992) and was, therefore, carried out despite one‐fourth of the variables being non‐normally distributed (revealed using the Shapiro–Wilks test). For these non‐normally distributed variables, follow‐up tests were conducted using non‐parametric alternatives (in this case, the Wilcoxon paired‐ranks test). Four follow‐up tests were conducted: two comparing differences between neighbouring and non‐neighbouring digits within tasks, and two comparing neighbours and non‐neighbours across tasks. Alpha levels were Bonferroni‐adjusted for these four comparisons (*p* < .0125).

#### 2.6.3 Multivariate representational structure

We used RSA (Kriegeskorte et al., 2008) to assess the multivariate relationships between the activity patterns generated across digits and tasks (Section 3.3). The (dis)similarity between activity patterns within the SI hand mask (based on BA1 and 3) was measured for each digit pair using the cross‐validated squared Mahalanobis distance (Nili et al., 2014). This was applied using the FSL‐compatible toolbox (D. Wesselink & Maimon‐Mor, 2017). First, the activity patterns were pre‐whitened using the residuals from the GLM, that is, noisy (groups of) voxels are downweighted, and then cross‐nobis distances were calculated for each task (active/passive) separately, using each pair of imaging runs and averaging those results. Greater distances indicate larger differences in multivariate representation; that is, the representations are less similar.

The above analysis produced 10 inter‐digit distance values per task, forming an RDM for each participant. We assessed two measures: (1) information content, which examines how different the activity pattern over voxels is for digit pairs on average. This was assessed by calculating the average value of the distances in an RDM (i.e., averaging across the values in the RDM, discounting values on the diagonal) and (2) representational structure, which looks at the pattern of inter‐digit distances across digit pairs. Specifically, we looked at how well the RDM of one individual correlated with the average RDM of all other participants (excluding the participant of interest). This was done within‐task (e.g., participant_X_ active and group mean active) and between‐task (e.g., participant_X_ passive and group mean passive). This allowed us to determine whether representational structure of the hand was more similar for individuals within a task versus between tasks. Note: RDMs were normalised prior to averaging to not bias the mean towards more dissimilar RDMs.

As an aid to visualise the RDMs (and not used in any statistical analysis), we also performed multidimensional scaling (MDS). This analysis projects the higher‐dimensional RDM into a lower‐dimensional space while preserving the inter‐digit distances as accurately as possible (Borg & Groenen, 2003). These plots allow multivariate inter‐digit relationships to be visualised spatially, for example, digits with more similar representations across voxels are projected as spatially closer together. MDS was performed on individual RDMs and averaged after Procrustes alignment (without scaling) to remove any arbitrary rotations introduced by MDS. Alignment was based on RDMs including both active and passive tasks (as well as a rest condition) as not to bias the visualisation. Dimensions were sorted on the basis of high variance between digits to demonstrate hand representation at the expense of non‐digit‐specific variation from rest. That is, the dimensions chosen for the X and Y axis were selected to best reflect (in terms of explained variance) inter‐digit differences, rather than to visualise the differences of the digits from rest.

Post hoc Bayesian analyses were carried out where non‐significant results were found for key comparisons using JASP (Version 0.16.2; using as a prior a Cauchy distribution centred on 0 with a width of .707). This was to provide further information on the strength of these null results and improve interpretation for the reader.

#### 2.6.4 Computational modelling

An exploratory post hoc computational model was used to investigate potential factors underlying the differences observed between tasks (see Supplementary Materials, Section 4, for more details). The TouchSim model (Saal et al., 2017) was used to generate the cutaneous passive peripheral inputs and reconstruct the typical activation of the different afferent classes across the hand. Specifically, SI is modelled with five units (representing the five cortical digit‐selective clusters) that receive input from the periphery and are also connected laterally, with each cluster exciting or inhibiting other clusters (Figure S4a,b). The parameters of this model were previously fit based on the passive task dataset (D. B. Wesselink et al., 2022), in order to replicate the cortical responses to passive digit stimulation from the univariate task data.

We tested whether simple input changes in the model, informed by basic physiology and biomechanics, might explain the observed changes in the active task. In particular, we first tested whether simply more overall activity (gain modulation) could explain differences between tasks by adding a gain parameter for the active task. Second, we tested whether digit enslavement patterns during the active task could account for differences between tasks by increasing the pooled input for each digit based on typical enslavement patterns during active movements (as measured in Ejaz et al., 2015, see Figure S6a left panel).

## 3 RESULTS

The performance of participants inside the scanner showed that participants followed the instructions well. During the active task, in 94.6% of the trials the target digit (i.e., the digit participants had been instructed to move) produced the strongest press force (92.2% in the worst participant). Consequently, there was a clear difference in average force output for the target (1.44 N, ±0.09 SEM) and non‐target (0.27 N, ±0.03) digits. To promote engagement in the passive task, participants were instructed to identify occasional ‘catch’ trials (double instead of single taps) that occurred once per digit/scan. Participants correctly identified the catch trials in 94.3% of the cases and there was no significant difference between digits (*F*(4, 70) = 1.85, *p* = .129).

### 3.1 Spatial correspondence is observed between tasks

To quantify the extent of spatial correspondence between digit maps across the active and passive tasks, we calculated the Dice coefficient (Dice, 1945). This coefficient was calculated across tasks and digits, resulting in a 5 × 5 matrix (see Figure 2b). Complete spatial overlap of two representations is indicated by a Dice value of 1 and no overlap by 0. Spatial overlap was maximal for comparison of the same digit between tasks (mean Dice = 0.68; see Figure 2c), compared with neighbouring digits between tasks (mean Dice = 0.32, *t*(14) = 16.22, *p* < .001). Moreover, neighbouring digits showed greater overlap between tasks than non‐neighbouring digits (mean Dice = 0.08, *t*(14) = 12.59, *p* < .001). Please see Supplementary Materials, Section 2 and Figure S3 for Dice values for the same digit within a task (split‐half analysis).

### 3.2 Consistency in somatotopic organisation between tasks

#### 3.2.1 Somatotopic index

We examined whether the classical hallmark of S1 organisation, the somatotopic gradient, is manifested similarly following passive versus active stimulation by correlating an index of digit preference with position along the central sulcus using circular correlation (see Section 2.6.2). This did not result in a significant group difference between the passive and active task (mean *r*_passive_: .63; mean *r*_active_: .71; *t*(14) = .655, *p* = .523, BF = 1.86). Coarse somatotopic organisation was, therefore, not different between the two tasks.

#### 3.2.2 Neighbourhood relationships analysis

We next examined activity level profiles for different digits within and across our digit‐specific clusters to investigate whether the activity patterns followed similar somatotopic neighbourhood relationships between tasks. Note that this was conducted within digit‐specific clusters created using the independent localiser (see Section 2.5.2 and example in Figure 3a). As a reminder, the term ‘target digit’ refers to activity resulting from touch/movement of a single digit within its own digit‐specific cluster.

First, we wanted to confirm that digit ‘selectivity’—with respect to the identity of the digit‐specific cluster—was found for both tasks. Selectivity is defined here as maximal activity in response to touch/ movement of a target digit within its own digit‐specific cluster. This was generally identified for both the active (69/75 winner‐takes‐all ‘hits’) and passive tasks (70/75 hits; see Table 1).

**TABLE 1:** Proportion of participants showing digit selectivity within each digit selective cluster for both tasks. Note C (i.e., for C1‐5), stands for cluster

Digit‐selective cluster	Active	Passive
C1	15/15	15/15
C2	13/15	15/15
C3	13/15	14/15
C4	14/15	14/15
C5	14/15	12/15
Total	69/75	70/75

Second, we looked at the amount of activity produced by the tasks (averaged activity across all ROIs in the two tasks). We found activity levels were greater overall in the active task in comparison to the passive task, *t*(14) = 2.85, *p* = .013 (see Figure 3b).

**FIGURE 4:**
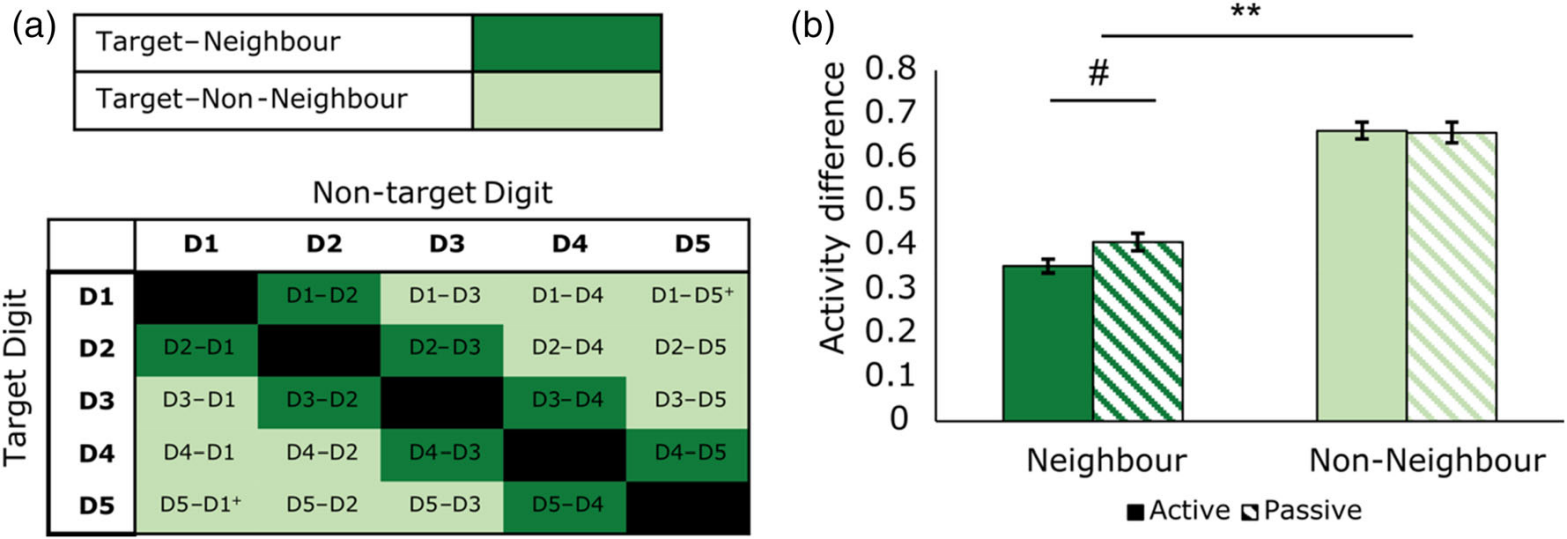
Comparing the neighbourhood relationships across the active and passive tasks. In this analysis, we examined these relationships between digits within the highly selective digit‐specific clusters defined by our independent localiser task. (a) Within each digit‐specific cluster, pairwise digit activity levels were contrasted. Each digit pair was characterised based on whether it contained a digit that was neighbouring or non‐neighbouring the target digit (dark green vs. light green bars). Results from this analysis are shown for the active and passive tasks in (b). For both tasks, the activity difference was greater between target digits and non‐neighbours than between target digits and neighbours, which is a hallmark of somatotopic mapping. We also found the activity difference between target digits and their neighbours is greater in the passive than active task, driving a significant interaction of neighbourhood × task. However, this difference did not survive correction for multiple comparisons. ** indicates significant differences at *p* < .001, # indicates a trend.

We then examined whether neighbourhood activity patterns were consistent between tasks. For both active and passive tasks, activity within each cluster generally appears to decrease as a function of distance from the target digit (Figure 3c,d). To examine these neighbourhood relationships statistically, we calculated the difference in activity between the target digit and its neighbours/non‐neighbours and divided this by the target digit activity level (Figure 4a). This resulted in difference values where larger values indicated a greater difference between target digit and the neighbour or non‐neighbour digits. A 2 × 2 repeated measure ANOVA was carried out with factors neighbourhood (neighbouring vs. non‐neighbouring digits), and task (active vs. passive task). This returned a significant main effect of neighbourhood (*F*(1, 14) = 650.89, *p* < .001), a non‐significant main effect of task (*F*(1, 14) = 1.50, *p* = .241) and a significant interaction of neighbourhood × task (*F*(1, 14) = 28.038, *p* < .001).

Two within‐task follow‐up Wilcoxon matched‐pair signed‐rank tests were conducted. Supporting somatotopy in these neighbourhood relationships, these tests showed that for both tasks the activity difference was greater between target digits and non‐neighbours than between target digits and neighbours (Active: *Z* = −3.408, *p* = .001, Passive: *Z* = ‐3.408, *p* = .001; see light green vs. dark green bars in Figure 4b). Two further follow‐up tests conducted between‐tasks demonstrated the interaction occurred because the activity difference between target digits and their neighbours was larger in the passive (mean passive difference = 0.41) than in the active task (mean active difference = 0.35); *Z* = −2.158, *p* = .031 (see dark green bars in Figure 4b). Note: this difference becomes non‐significant when Bonferroni corrections for four *t* tests are performed (adjusted *p* = .124). The activity difference between the target digit and non‐neighbouring digits was not different between the two tasks (mean passive difference = 0.66; mean active difference = 0.66; *Z* = −0.227, *p* = .820).

In sum, in both the active and passive task there was evidence of somatotopy in these neighbourhood relationships. The passive task, however, produced a slightly more pronounced somatotopic selectivity, though this difference held only for difference between target digits and their neighbours and did not survive corrections for multiple comparisons.

### 3.3 Multivariate representational structure is comparable between tasks but information content is higher in the active task

**FIGURE 5:**
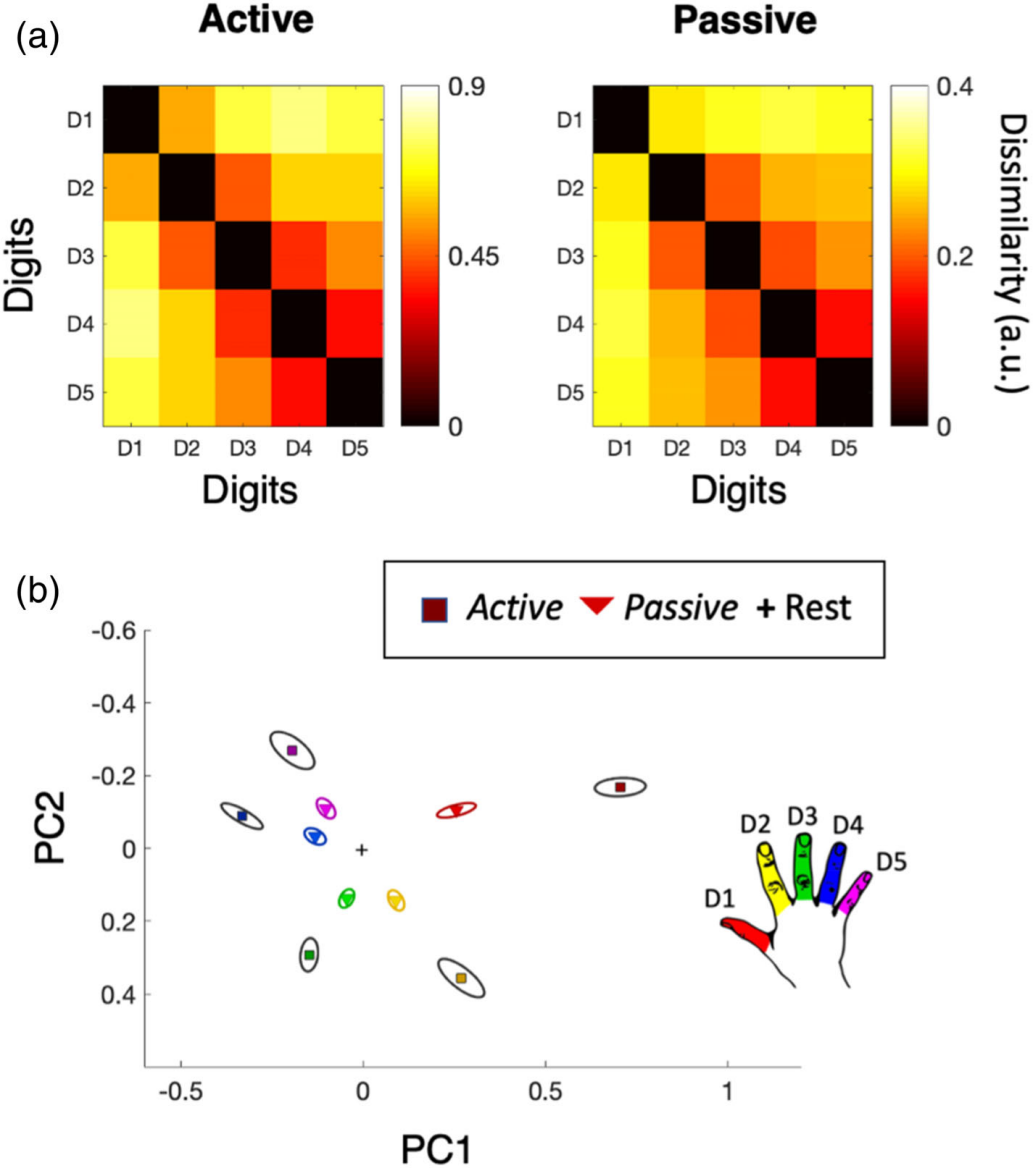
Multivariate hand representation during active and passive tasks. (a) Group mean representational dissimilarity matrices (RDMs) for the active (left) and passive (right) tasks. The colour bars indicate dissimilarity, such that hotter colours indicate greater dissimilarity across digit pairs. Please note, different scales have been used for the active and passive RDMs to allow comparability of the pattern of inter‐digit relationships which is obscured when the same scale is used (as the active task produced significantly greater dissimilarity overall, see Section 3.3). (b) 2D depiction of the data in (a), using multidimensional scaling (MDS). MDS projects the higher‐dimensional RDM into a lower‐dimensional space while preserving the inter‐digit distances as accurately as possible (smaller distances indicates more similar representations). Ellipses indicate between‐participant standard error, calculated separately per task. Red = D1, yellow = D2, green = D3, blue = D4, and pink = D5, black ellipses represent the active task, whereas coloured ellipses represent the passive task. Note: MDS plots are purely for visualisation purposes and were not used for statistical analysis. PCs: Principal components maximising inter‐digit dissimilarity.

Next, we used RSA to examine the multivariate representational structure of the hand (RSA; Ejaz et al., 2015; Nili et al., 2014). Figure 5a shows the inter‐digit distance matrices (RDMs) calculated for the active and passive tasks, based on the (unthresholded) activity patterns across the entire SI hand mask. Greater distances indicate larger differences in multivariate representation.

We first compared the overall strength of information content between tasks. This was examined by comparing the mean distances across all digit‐pairs (i.e., across each matrix) between tasks. We found mean distance was significantly greater for the active than the passive task, *t*(13) = 10.9, *p* < .001. As shown in Figure 5b, this means that on average each digit is represented more independently of the others (illustrated with greater physical distances) in the active task (see modelling of this result in Supplementary Materials, Section 4).

We next looked at whether the shape of the multivariate hand representation is consistent between tasks. This was achieved by first calculating within‐task correlation coefficients between each individual participant's RDM and the group average RDM obtained by averaging the RDMs from all other participants for that task. This was done for both the active and the passive task and resulted in two correlation coefficient values (one for each task) for each participant reflecting the correlation strength between that individual participants RDM and the group average RDM. We then compared these within‐task correlation coefficients across tasks and found no significant differences (*t*(13) = 0.16, *p* = .873; Bayes Factor_10_ = .273), ‘substantial evidence’ for H1 (Wetzels et al., 2011) between the correlation of individual participants' active RDM and the active group RDM (mean rho = .90), and the correlation of individual participants passive RDM and the passive group RDM (mean rho = .91; see Table 2). This indicates that for both tasks, individuals are similarly consistent with respect to the group's mean.

**TABLE 2:** Within‐ and between‐task correlation values and associated *t* tests comparing these correlation values. BF = Bayes Factor for the alternative hypothesis (H1) over the null (H0), that is, BF_10_

Between‐task correlations	Within‐task correlations	
	Active with active mean (mean rho = 0.90)	Passive with passive mean (mean rho = 0.91)
Active with passive mean (mean rho = 0.89)	*t*(14) = 0.89; *p* = .388; BF = .370	*t*(13) = 0.39; *p* = .706; BF = .288
Passive with active mean (mean rho = 0.90)	*t*(13) = 0.21; *p* = .834; BF = .275	*t*(13) = 0.69; *p* = .503; BF = .332

Next, we looked at the between‐task correlations. If both tasks evoke a distinct representational structure, between‐task correlations should be lower than within‐task correlations. However, the correlations were high for individual participants active RDMs with the passive mean RDM (mean rho = 0.89), and for individual passive RDMs with the active mean RDM (mean rho = 0.90). As demonstrated in Table 2, the within‐task correlations were not significantly different to the between‐task correlations (assessed by paired *t* tests; .388 < *p*'s < .834). Thus, the structure of the RDM is relatively stable independent of task demands.

### 3.4 Post hoc computational modelling

A computational model was used in order to explore factors which may have contributed to the differences we observed between tasks. Specifically, we explored whether simple scaling of activity (gain modulation) or whether digit enslavement patterns during the active task could explain differences between tasks. Results are summarised here, but due to the limited explanatory power of our model, the full discussion of the results are included in the Supplementary Materials, Section 4.

For the observed differences in univariate activity between the active and passive tasks, we found that neither global scaling, nor typical enslavement patterns seen in active tasks, could explain the differences observed between active and passive tasks. Rather, more complex digit specific changes were required at either the input level or at the cortical level to capture the differences across tasks. Specifically, during the active task, inputs from the thumb were reduced, whereas increased inputs were required for digits 2 and 3 for all digit stimulations (see Supplementary Figure S6b). These digit specific changes could not be attributed to a single hypothesised factor; however, the digit specific changes were able to reproduce the shrinking of the passive RSA dissimilarity compared with the active.

Finally, the model was able to explain differences between the active and the passive task RSA analysis based on a simple gain modulation effect (Figure S6c), suggesting that differences between tasks observed in this analysis are due to a simple scaling effect.

## 4 DISCUSSION

Here, we compared digit activity created by two distinct tasks, performed by the same group of participants: one involving passive cutaneous stimulation of the distal digit pad, and the other involving whole digit active movements. We examined how these two tasks affected fundamental spatial features of somatotopy (spatial correspondence of digit maps and somatotopic activity), as well as non‐spatial inter‐digit representational features (as revealed by multivariate analysis) in somatosensory cortex. We found that despite marked differences between afferent input, efferent output and top‐down factors, the activity patterns generated were largely consistent between these two tasks in SI. Specifically, we showed that the digit maps produced by active and passive tasks were largely spatially overlapping, both relative to somatotopy and in absolute position. Further, active and passive tasks both produce maximal activity to the stimulated digit within the stimulated digit's own ROI and a clear somatotopic pattern of activity with respect to its neighbours and non‐neighbours. Finally, we showed there was a strong correlation between the multivariate hand patterns produced in the active and passive tasks, indicating high‐level features of hand representation are highly similar between tasks.

Notwithstanding the preservation of these critical features at both the macro‐level (somatotopic map), and the meso‐structure of fine‐grained features (multivariate analysis), we also identified some notable differences between active and passive tasks. Specifically, consistent with previous reports (Berlot et al., 2019; Wiestler et al., 2011), our univariate analysis indicated the active task produced a greater overall amount of activity compared to the passive task. In the active task, we also found the activity patterns generated by different digits were more clearly distinguishable overall. That is, on average there was greater separability of the multivariate activity patterns for all pairings of digits in the active than passive task, that is, higher information content (see below for more discussion). Nevertheless, our univariate activity analysis produced some evidence suggesting greater activity differences between target digits and their neighbours (i.e., greater selectivity) in the passive task, though this effect was limited and did not extend to the non‐neighbouring digits. Together, this suggests that while information content was overall greater in the active task, there were still some representational features that were more prominently identified in the passive task using univariate selectivity. Therefore, our findings highlight that multivariate and univariate activity metrics represent different aspects of neural digit representation, and the nuance of what each analysis uniquely conveys should be considered when interpreting their results.

### 4.1 Task differences in SI plasticity research

Historically, there has been speculation that the use of different tactile stimulation protocols could contribute to some differences in somatotopic maps identified between different studies, though largely without formal substantiation. For example, in the field of human brain remapping following amputation, research using passive stimulation to the lower face has documented massive shifts of facial representation into the missing hand cortex (Flor et al., 1995); though see (Valyear et al., 2020). Other paradigms using active facial movements indicate relatively stable representation, with little activity in the missing hand area from facial movements (Kikkert et al., 2018; Tamar R. Makin et al., 2013; Root et al., 2021), and shifts of representation documented only locally within the face area (T. R. Makin et al., 2015; Raffin et al., 2016). Previous studies attempted to bridge these noticeable gaps by matching across task demands as closely as possible (Berlot et al., 2019). Here, by using tasks not tightly matched in stimulation features, we allow stimulation to vary between tasks in a manner more typical of, for example, variation between studies (Dempsey‐Jones et al., 2019; Kuehn et al., 2014; Martuzzi et al., 2014; Striem‐Amit et al., 2018; Yu et al., 2019). Our results provide direct evidence to demonstrate that active and passive tasks elicit spatially overlapping somatotopies in typically developed individuals.

The between‐task consistency reported here is further supported by clinical plasticity studies that reveal similar patterns of sensorimotor reorganisation across active (Foell et al., 2014; Lotze et al., 1999) and passive (Flor et al., 1995; Karl et al., 2001) stimulation paradigms. For example, Striem‐Amit et al. (2018) previously studied sensorimotor somatotopy of individuals born with no hands due to congenital upper limb malformation. The authors reported similar inter‐body part remapping using both active and passive stimulation. Together with our current findings, this evidence indicates that both neurotypical somatotopic organisation and remapping are qualitatively and quantitatively similar when expressed across active and passive tasks. Given that the mechanisms of activation are distinct, however, we cannot rule out the possibility that active and passive tasks might cause differing representations under certain clinical conditions; for example, in motor neuron disorder where there are impairments in efferent outflow with largely maintained afferent input.

Importantly, we note that while the spatial overlap between the active and passive maps was high, it was not complete (see Supplementary Materials, Section 2). It is also important to consider that our methodology might not be sufficiently sensitive to identify other representational motifs that may vary across the tasks. For example, laminar differences may be more readily identifiable using other recording techniques than gradient echo imaging (as here), which biases towards activity in superficial layers (Goense et al., 2012). Despite this, our acquisition methodology represents the state of the art for fMRI, and therefore, our study provides a very reasonable benchmark for the level of evidence acquired in neuroimaging studies. Insofar as most of the representational features we identified did not vary between tasks, we believe our findings challenge arguments that differences in stimulation type are the sole cause of large‐scale differences in reported (re)organisation of the body map.

### 4.2 Mechanisms underpinning representational consistency between tasks

While the bottom‐up and top‐down differences between tasks may lead one to expect considerable differences in SI representation, there are several neurophysiological mechanisms that could support the representational consistency we report here. For example, it is known that somatosensory inputs feed into morphologically defined digit regions, particularly in BA1 and 3 where somatotopy is most pronounced. Extensive work in primates demonstrates the existence of myelin‐poor regions (‘septa’) between myelin‐rich representations of major body parts at cortical (e.g., Jain et al., 1998; Qi & Kaas, 2004) and subcortical levels (e.g., Kaas et al., 1984). Various lines of evidence suggest septa create borders between neurons that strongly interact and neurons that weakly interact (Hickmott & Merzenich, 2002). Accordingly, septum is best observed at the hand‐face border (now documented in humans with fMRI: Kuehn et al., 2017), but also present, though to a reduced extent, between the digits (in primate area 3b: Jain et al., 1998; Qi & Kaas, 2004). Thus, if somatosensory inputs from the active and passive tasks both feed into similar morphologically defined regions in SI, this could contribute to similar patterns of hand representation between tasks.

Indeed, the existence of precise somatotopic feedforward projections throughout the somatosensory system could underlie general consistency in the eventual hand representation produced in SI. The majority of inputs to SI come from the dorsal column system, the largest somatic pathway of the spine (ten Dokelaar, 2002). The dorsal column is known to consist of somatotopically organised ascending fibres (Walker & Weaver, 1942). Consequently, tactile inputs feed from specific digits in the periphery, to the matching digit‐specific areas in the cuneate (Culberson & Brushart, 1989; Florence et al., 1989), thalamus (Kaas et al., 1984) and early SI (Kaas et al., 1979). Variation does exist in the decomposition and path of somatic information from different mechanoreceptor systems from the periphery to SI (Bensmaia et al., 2008). However, on the whole, the preservation of somatotopy from receptors to central processing regions could support the generation of similar representations of the digits from active and passive tasks in SI.

Related to the above, under most conditions, active tasks can be expected to activate a different or wider range of peripheral receptors than passive tasks (discussed further below). While divergent inputs to SI of this kind could feasibly cause large differences in SI digit‐representations, animal work suggests different types of input to SI, for example, from different mechanoreceptor classes, feed into highly interspersed modular zones in SI, that is, cortical columns (Chen et al., 2001; Friedman et al., 2004; Mountcastle, 1957). For example, vibrational stimuli in the tap, flutter and vibration ranges activate spatially separate columns in areas 3b (Chen et al., 2001) and 1 (Friedman et al., 2004) of around 200 μm in diameter. These columns appear to be arranged in a continuous, pinwheel organisation, as with orientation columns in the visual system (Yacoub et al., 2008). While differences in columnar activation may occur between our tasks at the micro‐level, this could still result in similar macro‐level representation of the digits, as is derived from traditional fMRI measures, even at the high imaging resolution we use here. Thus, as discussed above, while different measures may elucidate task differences in SI, our study is able to speak to our aim of exploring whether hallmark features of SI hand representation, as commonly measured with fMRI, vary under active and passive tasks.

As mentioned in Section 1, somatosensory cortex contains direct projections to the body. For example, Karadimas et al. (2020) demonstrated that stimulating corticospinal projections from somatosensory cortex to cervical excitatory neurons can increase locomotion, while inhibition can decrease or terminate locomotion. Research such as this raises the interesting, if speculative, idea that the SI digit maps generated by active movement that we present here contain both sensory and motor components. In comparison, SI digit maps resulting from our passive task should represent sensory only components. Comparing digit representation under our active and passive tasks, therefore, allow us to compare sensory + motor (active) versus sensory (passive) representations in somatosensory cortex, respectively. While it would be interesting to examine the motor only versus sensory only aspects of digit representation in SI, it is difficult to study in healthy human participants and fMRI.

### 4.3 What explains the between‐task differences observed?

Despite general consistency in various features of digit representation tested, we also identified some differences between tasks. Most notably, we found significantly more overall activity in the active task. This could be the result of the greater number and range of afferent, efferent and top‐down inputs associated with active movement. Even solely considering cutaneous afference, active tasks tend to produce more forceful indentations of the tips of the digits than light touch, air puffs or electrostimulation. A deeper indentation leading to more widespread mechanoreceptor activation, and a general change in the volume of the digit as the key was pressed would likely lead to more complex and widespread central activity resulting from the active task (Saal et al., 2017). Active tasks may also cause more activation of skin stretch and proprioceptors in the joints during movement (Bensmaia & Miller, 2014; Chouvardas et al., 2008; Saal et al., 2017), leading to greater activity overall. It is also likely that reward (Pleger et al., 2008) and attentional effects (Puckett et al., 2017), known to modulate digit representations in SI, contribute to activity differences seen here between passive stimulation and active movement. While in our study, we cannot delineate whether or how top‐down and bottom‐up contributions to this activity difference, Berlot et al. (2019) tightly matched task and simulation properties, and also reported greater overall activity in active compared to passive tasks. This may suggest a greater role for efference and motor contributions to creating this difference, than other sensory or top‐down differences.

We also found higher information content in the active task, as reflected in higher multivariate dissimilarity values overall. This could also be caused by the greater amount and variety of sensory inputs produced by active tasks, which could further improve the signal‐to‐noise ratio and, in turn, afford improved discriminability between digits. Paradoxically, it could also be the fact that individual digits are limited in their ability to move independently (‘enslaving’; Lang & Schieber, 2004) that could improve discriminability under active tasks. As most SI input results from active touch, sensory information produced by the active task is more aligned with typical, ecologically relevant patterns of sensory input. Indeed, while early SI areas (particularly 3b) contain predominantly neurons with single‐digit receptive fields, it is being increasingly recognised they also contain considerable numbers of neurons with complex, multi‐digit receptive field structures (Iwamura et al., 1994; Thakur et al., 2012), like those that predominate in later somatosensory areas (such as BA 1 and 2; reviewed in Iwamura et al., 2002). Considering that in daily life, single digits are rarely tactually targeted in complete isolation and active movement likely involves more complex inputs from across the hand, this may produce a more optimal input to these multi‐digit RFs. This could, in turn, lead to greater dissimilarity between digit representation in the active task. In this context, we wish to emphasise that we did not find a clear contribution of digit co‐movement (e.g., due to enslavement) in the active task to our findings. This is demonstrated implicitly in the strong correlation of multivariate representational structures between tasks, but also explicitly in our computational model. Instead, inter‐digit co‐use might become a fundamental aspect of digit representation (see Ejaz et al., 2015 for the mechanism of this overlap as it pertains to the natural statistics of action). Further, evidence suggests a key role for central mechanisms in generating motor enslavement; thus, co‐activation patterns are likely to hold even in the absence of large overt movements (as with our active task). Please note, while it is possible some of the co‐activation seen in the active task (e.g., high activity levels following stimulation of digit four and five within cluster four) could be attributed to co‐movement of these two digits in the active task due to enslaving (Lang & Schieber, 2004), a highly similar pattern of co‐activation is seen in the passive task (no movement). This suggests these co‐activity patterns cannot be attributed to motor enslaving during the task alone, but rather likely represent a fundamental aspect of digit representation, for example, the overlap of digit four and five representations (see Ejaz et al., 2015 for the mechanism of this overlap as it pertains to the natural statistics of action).

As stated previously, after identifying these task differences, we used a computational model of somatosensory cortical responses (previously described in Wesselink et al., 2022), to explore two key mechanistic factors that could give rise to the differences observed, gain modulation and digit enslavement patterns. The modelling revealed that simple scaling (gain modulation) could account for the differences between active and passive tasks as reflected in the multivariate data. In contrast, gain modulation did not describe the differences between tasks as seen in the univariate data. Digit enslavement, a key input difference between active and passive tasks, also did not account for the univariate task differences seen. Instead, more complex digit specific changes at the input or the cortical level were required to capture the differences across tasks on the univariate analysis which could not be attributed to a single hypothesised factor. In other words, more research in needed in order to capture and interpret the subtle somatotopic differences between active and passive tasks.

## 5 LIMITATIONS

In this study, we wished to investigate whether somatotopic and representational structures are shared across active and passive tasks, when stimulus features are not tightly matched. It should be noted, however, that not matching stimulus features does limit interpretation of the differences observed. For example, the increased activity observed in the active task could be due to several factors that varied between the tasks including the force, efference copies, reward, attentional demands, or the reduced amount of overall somatosensory stimulation provided in the passive task. Further, differences in constant vs. periodic sensory input would have differentially engaged rapidly and slowly adapting mechanoreceptors (Johnson, 2001; Vallbo & Hagbarth, 1968) and led to differing levels of peripheral adaptation (Klocker et al., 2016). Given the current study focused on whether, in spite of these differences, general consistency in hand representation could be found (rather than parametrically determining how specific stimulus features affect the hand map), this was not considered to invalidate our experimental aims.

## 6 CONCLUSIONS

Our study suggests active and passive tasks may be similarly used for exploring the somatosensory hand representation using fMRI. This has practical considerations for the implementation of studies on the ground—as active tasks can be easier to set up and may not require as much specialist equipment. However, it should be highlighted that decisions surrounding the use of active or passive task paradigms should always be informed by the scientific question to be addressed. Whilst active tasks may provide more ecologically valid input to the somatosensory cortex, passive paradigms may be superior in situations where motor demands cannot be reliably matched. It has further practical implications for situations where either active or passive stimulation may not be possible: such as in people with limited mobility, or amputees who are missing physical digits but can produce active movements in their phantoms (Kikkert et al., 2016). On a more fundamental level, our findings have deeper implications for our understanding of SI, as the product of a tight, and bidirectional relationship with the motor system.

## AUTHOR CONTRIBUTIONS

Zeena‐Britt Sanders, Harriet Dempsey‐Jones, Daan B. Wesselink, and Tamar R. Makin conceptualised the study, developed/designed the methodology, were responsible for project administration. Zeena‐Britt Sanders and Daan B. Wesselink did data curation and visualised data. Zeena‐Britt Sanders, Daan B. Wesselink, and Laura R. Edmondson performed the formal analysis. Zeena‐Britt Sanders, Daan B. Wesselink, Laura R. Edmondson, and Hannes P. Saal programmed the code/software. Zeena‐Britt Sanders, Daan B. Wesselink, and Harriet Dempsey‐Jones performed the investigation (data collection). Zeena‐Britt Sanders, Harriet Dempsey‐Jones, and Tamar R. Makin wrote the original manuscript. Alexander M. Puckett, Hannes P. Saal, and Tamar R. Makin provided supervision/mentorship. Tamar R. Makin acquired financial support for the project.

## FUNDING INFORMATION

TRM was funded by a Wellcome Trust Senior Research Fellowship (https://wellcome.ac.uk/, grant number: 215575/Z/19/Z) and by the European Research Council (https://erc.europa.eu/, 715022 Embodied Tech). LRE and HPS were funded by a Leverhulme Trust Research Project Grant (RPG‐2022‐031). The authors also received funds from Medical Research Council (MC_UU_00030/10).

## CONFLICT OF INTEREST

The authors declare no financial/nonfinancial conflicts of interest.

## Supporting information


**APPENDIX S1**. Supplementary Information

## Data Availability

The data that support the findings of this study are openly available in the Open Science Framework at https://osf.io/nh4yp/, reference ‘Malleability of the cortical hand map following a finger nerve block.’
