# Supplementary material for: Similar somatotopy for active and passive digit representation in primary somatosensory cortex
Source: Hum Brain Mapp. 2023 May 5;44(9):3568–85. doi: 10.1002/hbm.26298 (PMC10203813; doi:10.1002/hbm.26298)
Supplement: Supplementary file 1 — APPENDIX S1. Supplementary Information [file HBM-44-3568-s001.docx]

**Supplementary Materials**

**
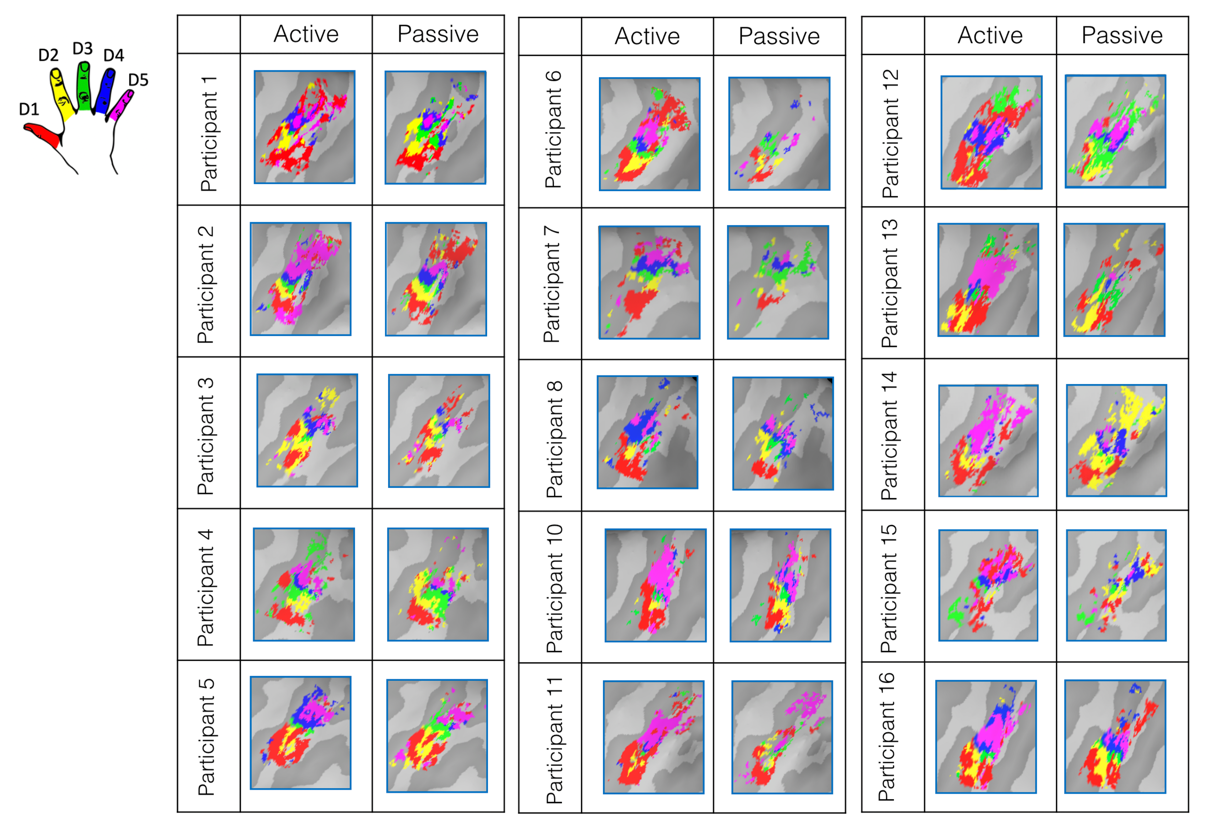
**

*Figure S1*: Minimally-thresholded activity (Z >2) within the SI hand mask of each digit across the active and passive task is shown for all participants. This data was used for the Dice analysis.

**1. Traditional Dice produces same results (refers to: 2.6.1 Spatial correspondence in main text)**

The traditional Dice analysis is calculated by dividing the amount of spatial overlap between, in this case, two digit representations, by the total spatial area. Due to the variance in spatial extent across tasks and across digits, for the main analysis we divided the overlap by the smallest digit representation area, therefore normalising overlap to the smallest digit area. However, we also conducted the traditional Dice analysis and results were largely the same, despite lower overlap values on the whole (see *Figure S2*). Dice values for the same digit across tasks decreased from 0.681 to 0.412, and for neighbouring digits from 0.315 to 0.179.

However, despite decreases in the average Dice coefficient values, the relationship between tasks and digit pairs remained the same. Meaning that across the tasks, the same digit produces higher spatial overlap than each digit compared to its neighbour t(14)=13.85,p<0.001 and compared to non-neighbouring digits t(14)=13.01, p<0.001. Therefore, results were the same using the traditional dice analysis as using the modified dice analysis presented in the main text.


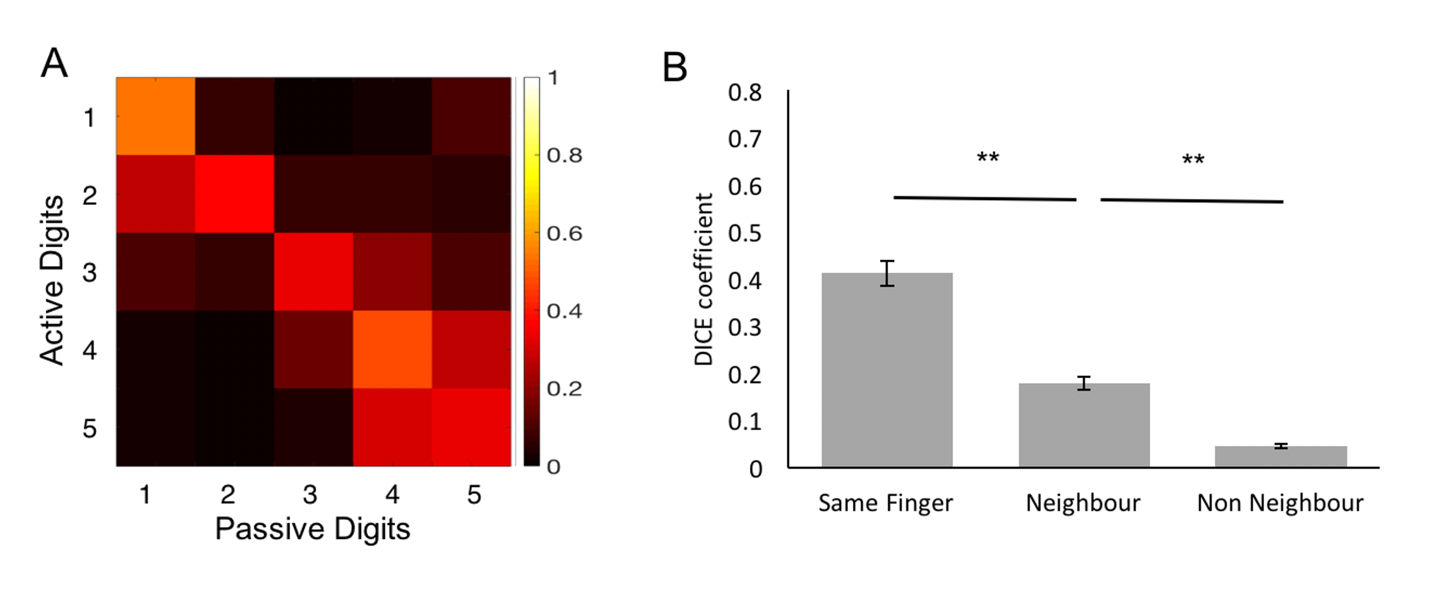


*Figure S2*: A) 5x5 matrix showing spatial overlap in SI hand mask as measured by the (non-normalised) dice coefficient between active and passive digits. The Dice coefficient shows greater overlap between homologous digits across tasks, as well as greater overlap between neighbouring than non-neighbouring digits. All other information is detailed as in Figure 2.

**2. Higher spatial overlap for same digit within tasks than between tasks (refers to 3.1 Spatial correspondence is observed between tasks in main text)**

DICE values were also calculated within tasks in order to assess how much variance can be expected to occur within tasks across runs. This was compared to the DICE values calculated between tasks. For the within task DICE a split-half consistency approach was used, in which data was split into odd and even runs for each task, and overlap between each digit pair across odd and even runs (e.g., odd D1 v. even D2) was calculated per task, and then averaged across tasks. To facilitate a fair comparison with the between task DICE in terms of the amount of data used for comparisons, a split-half approach was also used to compare between tasks (for example: odd Passive runs v. odd Active runs) and then averaged across runs. The average DICE value was then compared within and between tasks for the same digit, neighbouring digits and non-neighbouring digits using a Bonferroni corrected p-value (for three comparisons) of p=0.016. Spatial overlap was significantly higher for the same digit within task than between tasks (t(14)=3.96, p=0.001). Average DICE values were 0.68 for the within task comparison versus 0.61 for the between task comparison (see *Figure S3*). There were no significant differences when comparing the DICE values within and between tasks for neighbouring or non-neighbouring digits (neighbouring: t(14)=1.76, p=0.10, non-neighbouring: t(14)=-2.00, p=0.065).


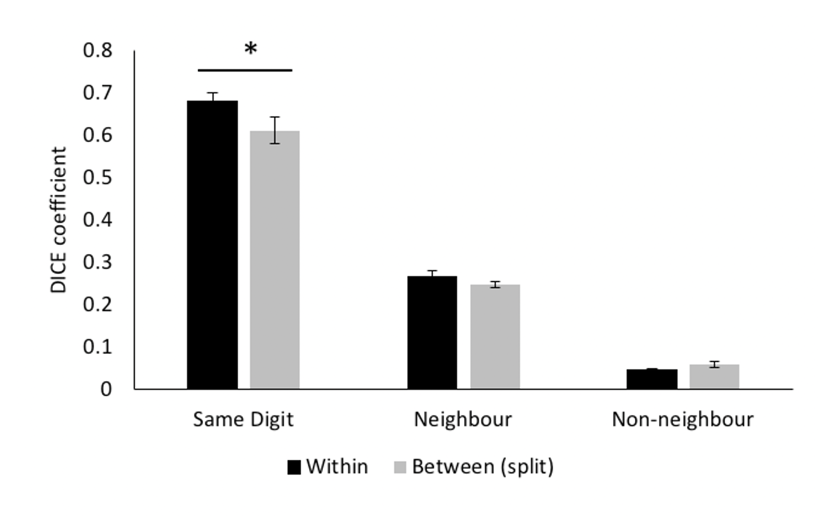


*Figure S3*: DICE coefficient is shown for the same digit, neighbouring digits and non-neighbouring digits both *within* task (black) and *between* tasks (grey). There is significantly greater spatial overlap for the same digits when compared *within* task. Error bars represent S.E.M. * p<0.016.

**3. Somatotopic gradients are maintained when including D5 as a neighbour to D1 (refers to 2.6.2 Somatotopic gradient of activity in main text)**

When analysing the somatotopic gradient within digit-specific clusters, in the main text D5 was treated as a non-neighbour to D1. However, double D1 representation (i.e., two completely/ partially spatially separated areas representing D1) has been documented previously (Kikkert et al., 2016) and was observed in the current study in both the active and passive task. When conducting the same analysis as described in section 2.6.2 but including D5 as a neighbour to D1 (instead of as a non-neighbour), the main results remained largely unchanged. The 2x2 ANOVA still showed a significant interaction between task and neighbourhood F(14,1)=38.864,p<0.001. Follow-up Wilcoxon related-samples signed-rank tests revealed that the differences between target and neighbouring digits was significantly smaller than differences between target and non-neighbouring digits for both active and passive tasks (both p=0.001). Additionally, the differences between target and neighbouring digits across tasks was also significantly different (Z=-2.499, p=0.012), with the passive condition having greater differences compared to active. Finally, the difference between target and non-neighbouring digits was not significantly different between the two conditions (Z=-0.284, p=0.776).

These results were all in line with the results reported in the main analysis, indicating that including D5 as a neighbour to D1 does not affect the results of this analysis.

**4. Computational modelling to investigate cortical and input factors that could underlie identified task differences (refers to 3.4 Post hoc computational modelling in main text)**

We aimed to investigate factors contributing to the differences observed between passive and active tasks using a mechanistic model of somatosensory cortical responses, previously described in Wesselink et al. (2022).

Cutaneous, passive peripheral tactile inputs were generated using the TouchSim model (Saal et al., 2017). This model reconstructs the typical activation of the different afferent classes across the hand (see *Figure S4A*).


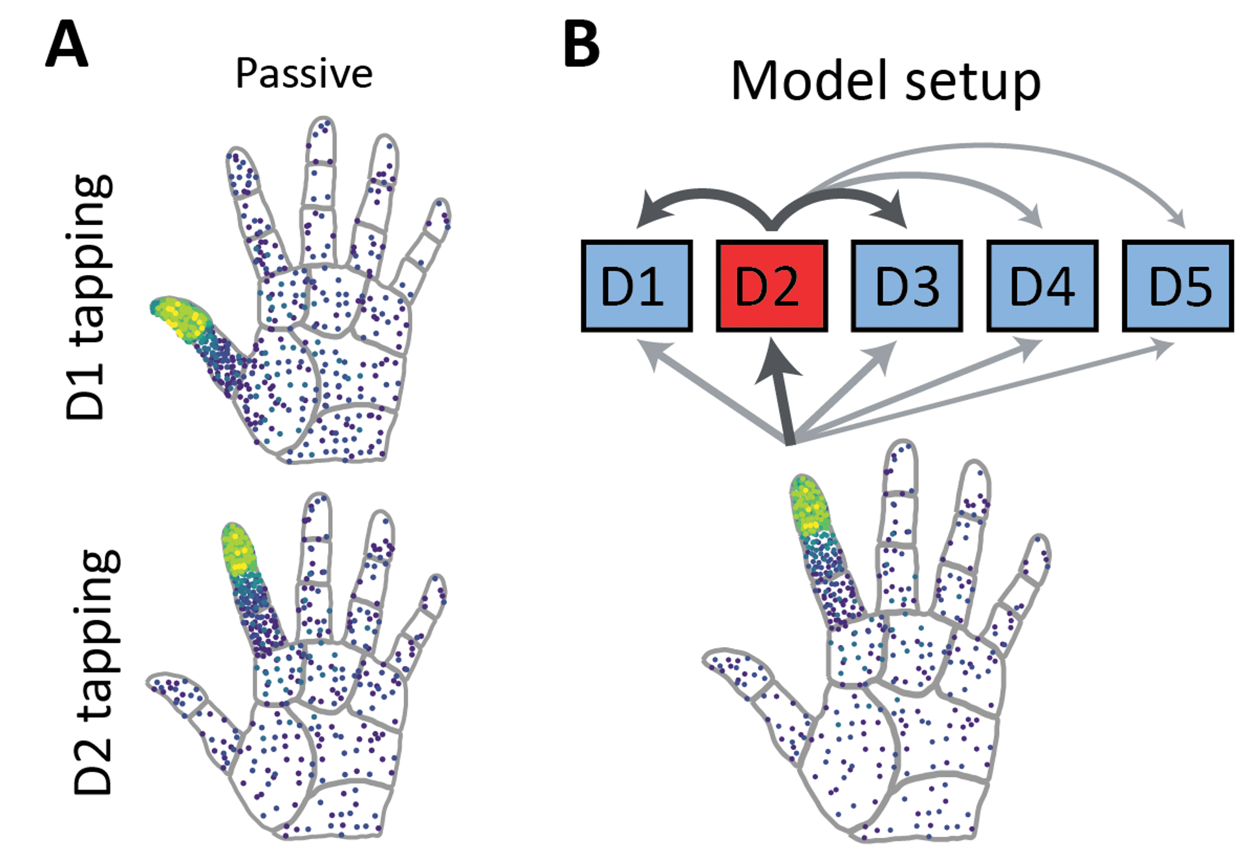


*Figure S4.* Computational model of cortical somatosensory responses to passive and active digit stimulation. (A) Modelled responses to passive stimulation of digit tips (examples for D1 and D2 stimulation shown). Lighter colours show higher firing rates of afferents. (B) Cortical model with five clusters, each representing a digit selective cluster corresponding to a digit. A set of feedforward weights connects each digit to each cortical unit. Clusters that correspond to a digit (e.g., D2 digit in cluster C2) are more strongly connected. Each cortical cluster is connected to all others through a set of lateral connections. The strength of these connections is denoted by arrow thickness, where neighbours are more strongly influenced.

The model was first fit to replicate the cortical responses to passive digit stimulation using the empirical univariate data. For the passive condition, inputs were modelled as the tapping of each of the five digits, mimicking the fMRI task. For each digit, the peripheral inputs were pooled. The cortical model consists of five clusters, broadly representing the preferred tuning of each of the five digits. The model has both feedforward and lateral connections between units (to account for input and cortical factors, respectively; see *Figure S4B*). Feedforward connections occur between the input digits and each cortical cluster (see *Figure S4B*). Lateral connections exist between the cortical clusters, with each cluster exciting or inhibiting other clusters. The strength of this is determined by the activation of each cluster under each digit’s stimulation. The lateral connection field is updated iteratively in the model until the cortical cluster activations settled (usually within six timesteps). For each timestep, *t*, a single-cluster activation is calculated according to the equation:

c_i_(t) = α_i_**w**_i_^T^**d** + b + **l**_i_^T^**c**(t-1)                   (1)

where *c_i_(t)* is the activation of cortical cluster *i* responses at timestep *t*, α is a gain factor, **w** is a weight vector specifying each digit’s connection strength to the cortical cluster, **d** is the vector of peripheral inputs from each digit, *b* is a scalar offset, **l** is the lateral connection field and **c**(t-1) is the vector of cortical activations from the previous timestep. The lateral connections depend only on the distance between clusters *i* and *j,* therefore the lateral pattern was the same for all clusters, only shifted spatially. The scalar offset, *b*, was increased so the typical pattern of lateral connectivity as shown in *Figure S4B* could be used. Both parameters **w**_i_ and *b* were fitted in the passive condition using multiple regression, with inputs from the TouchSim model. Weights were then adjusted using an iterative process to account for the dynamic lateral updates. As the initial cortical model was fit with passive inputs, all gain factors *α* for the passive case were set to 1.

*
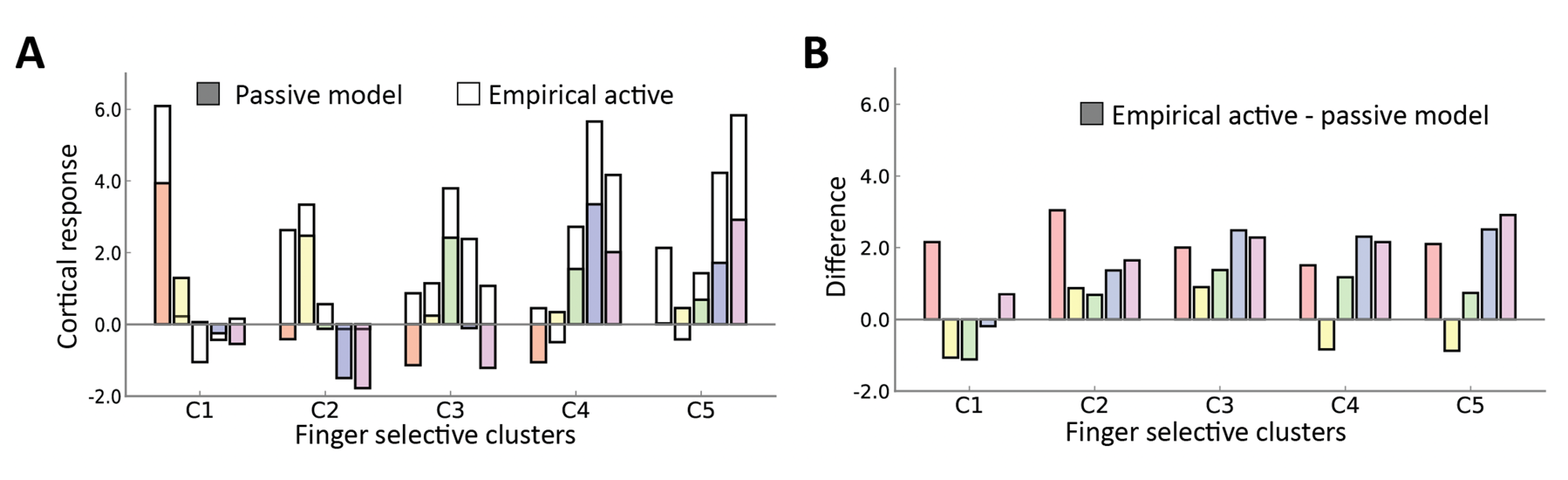
*Similar to the empirical univariate results, we calculated the activation of each cortical cluster to digit stimulation of individual digits in the passive case. We then compared the passive univariate model with the empirical univariate results for active stimulation, see *Figure S5A-B*. Next, we investigated whether simple changes at the cortical or input levels could account for the differences found between the passive model and empirical active data.

*Figure S5.* (A) Responses of the model digit-selective clusters (C1-C5) during stimulation of each digit. Model responses fitted on passive peripheral inputs are shown with coloured bars. The empirical univariate results for the active case are shown with white bars. (B) What changes are needed to fit the active empirical case? Here we show the differences between the passive model and active empirical results. A U-shape emerges for each digit cluster, whereby differences are larger for D1 and D5 than others.

Specifically, we first examined whether the pattern of activity over digits is consistent between active and passive conditions, with the active condition simply showing more activity overall (gain modulation). This could occur due to differences at the cortical level, as discussed in the main text. In this case, the *α* model gain parameter was varied for the active task to test whether a global gain change could account for the differences. Here, α was varied at the same rate for all clusters and digit stimulations.

Secondly, we examined a key input difference between the active and passive tasks, digit enslavement. Enslavement could increase co-activation of digits during active pressing, increasing cutaneous and/or proprioceptive inputs. Here we increased the pooled input for each digit based on typical enslavement patterns to account for this effect. The level of increase was based on previous data of digit enslavement patterns during active movement (keyboard presses, similar to those used in the current study) from Ejaz et al. (2015), see *Figure 6A*, left panel.


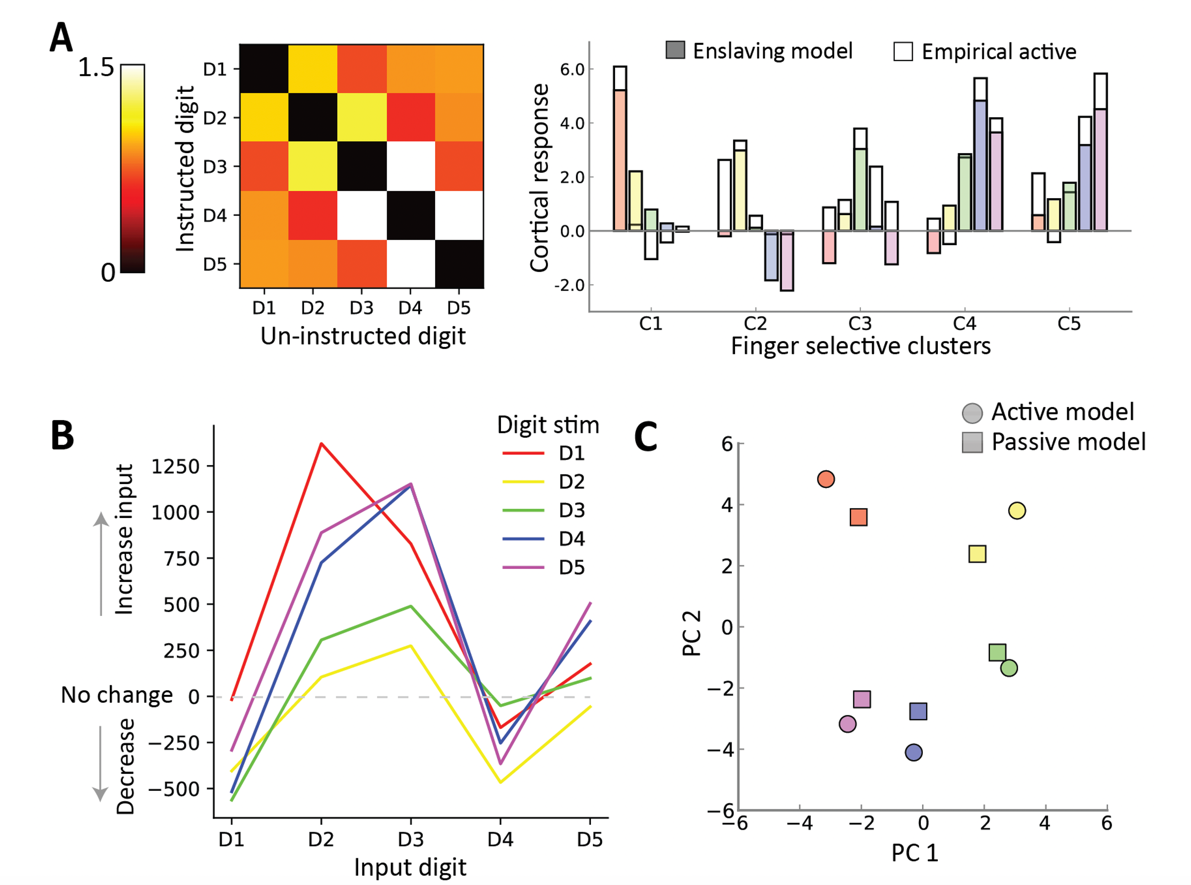


*Figure S6.* (A) In the active condition, additional afferent or proprioceptive inputs may occur through enslavement of digits. Beginning with the passive model, we increased the inputs for each digit stimulation using a matrix of previous data representing enslavement of digits in active movement (Ejaz et al., 2015; left panel). However, this was unable to accurately reproduce the pattern of active univariate responses (see right panel). (B) What changes are required at the input level in the passive model to reproduce the active case? Similar patterns of change are required for all digits, where the input for D2 and D3 is largely increased. The amount of required input increase is lower when either D2 or D3 are stimulated.(C) Using the pattern of input identified in B for the active case, we used RSA to calculate the dissimilarities of the model cluster responses to each digit input pattern. Multidimensional scaling of the dissimilarity matrix demonstrates the shrinking of passive stimulation compared to active.

In sum, looking at the univariate data, we found the activation differences between the passive model and empirical active data could not be explained with a simple global gain change for all digits at cortical level, such as a general scaling up or down of the activation. Further, implementing the typical enslavement patterns at the input level, by increasing tactile inputs from typically co-active digits, was also unable to reproduce the pattern of active activation alone.

Instead, we found digit specific changes were required at either the input level or at the cortical level (gain changes) to match the active empirical data to the passive model. The required digit specific changes in input are shown in *Figure S6B*. They demonstrate that under the active condition (compared to passive), inputs from the thumb were reduced, whilst digits D2 and D3 required increased input for all digit stimulations. The digit specific changes to the input level of the model, used to match the passive model to the active empirical data (univariate), was also able to reproduce the shrinking of the passive RSA dissimilarity versus the active RSA (see *Figure S6C)*, as also shown in the empirical data. Although the general pattern of input changes was similar for each digit stimulation condition and therefore appeared systematic (see *Figure S6B*), we were unable to identify a simple factor which could reproduce this pattern. It is therefore likely that multiple factors distinguish the active from the passive conditions, when looking at the univariate data. For example, afferent input changes may be coupled with cortical changes or inputs from other cortical regions. However, we are unable to disentangle the precise contribution of these influences in the current model and suggest that the differences between passive and active cortical activation warrant further investigation.

Unlike for the univariate data, simple scaling was enough to account for the differences between the passive RSA model and the active RSA empirical data. Therefore, as with the empirical data, the differences between active and passive conditions varied slightly depending on whether univariate or RSA methods were used.

**References**

Ejaz N, Hamada M and Diedrichsen J (2015) Hand use predicts the structure of representations in sensorimotor cortex. *Nature neuroscience* 18: 1034-1040.

Kikkert S, Kolasinski J, Jbabdi S, et al. (2016) Revealing the neural fingerprints of a missing hand. *Elife* 5.

Saal HP, Delhaye BP, Rayhaun BC, et al. (2017) Simulating tactile signals from the whole hand with millisecond precision. *Proc Natl Acad Sci U S A* 114(28): E5693-E5702.

Wesselink DB, Sanders ZB, Edmondson LR, et al. (2022) Malleability of the cortical hand map following a finger nerve block. *Sci Adv* 8(16): eabk2393.
